# Supplementary material for: Large-Scale Expansion of Human iPSC-Derived Skeletal Muscle Cells for Disease Modeling and Cell-Based Therapeutic Strategies
Source: Stem Cell Reports. 2018 May 3;10(6):1975–90. doi: 10.1016/j.stemcr.2018.04.002 (PMC5993675; doi:10.1016/j.stemcr.2018.04.002)
Supplement: Document S1. Supplemental Experimental Procedures, Figures S1–S3, and Tables S1–S4 [file mmc1.pdf]

Stem Cell Reports, Volume *10*

Supplemental Information

Large-Scale Expansion of Human iPSC-Derived Skeletal Muscle Cells for Disease Modeling and Cell-Based Therapeutic Strategies

Erik van der Wal, Pablo Herrero-Hernandez, Raymond Wan, Mike Broeders, Stijn L.M. in 't Groen, Tom J.M. van Gestel, Wilfred F.J. van IJcken, Tom H. Cheung, Ans T. van der Ploeg, Gerben J. Schaaf, and W.W.M. Pim Pijnappel

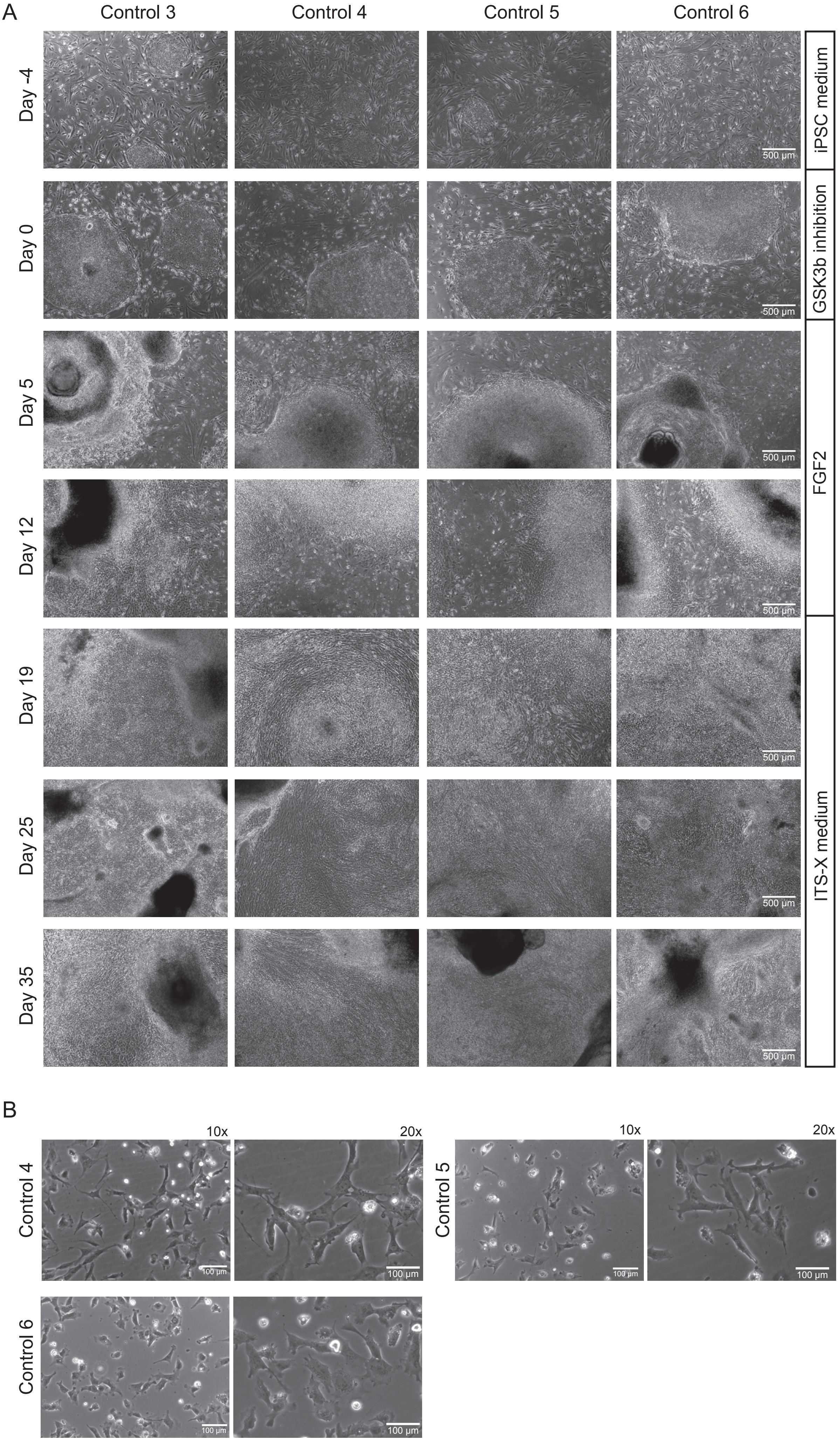
A
Control 3
Control 4
Control 5
Control 6
Day -4
Day 0
Day 5
Day 12
Day 19
Day 25
Day 35
iPSC medium
GSK3b inhibition
FGF2
ITS-X medium
500 µm
B
10x
20x
Control 4
Control 5
Control 6
100 µm

Figure S1

A

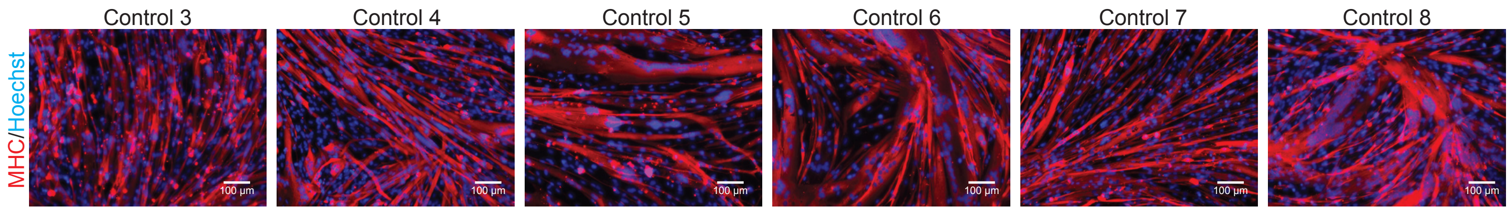

	Control 3 average	Control 3 sd	Control 4 average	Control 4 sd	Control 5 average	Control 5 sd	Control 6 average	Control 6 sd	Control 7 average	Control 7 sd	Control 8 average	Control 8 sd
**5-14d**	59,1%	5,3%	51,4%	3,9%	52,9%	7,0%	39,2%	11,8%	63,1%	1,7%	91,6%	4,4%
**12-18d**	69,4%	4,8%	37,3%	1,4%	37,9%	12,1%	40,1%	11,1%	51,8%	14,4%	62,7%	13,4%
**20-25d**	54,3%	16,2%	46,0%	8,6%	57,9%	7,8%	81,9%	6,3%	48,4%	20,5%	50,8%	2,3%
**27-35d**	31,4%	6,8%	49,5%	7,0%	64,1%	3,6%	79,6%	4,0%	35,3%	1,7%	41,7%	4,3%

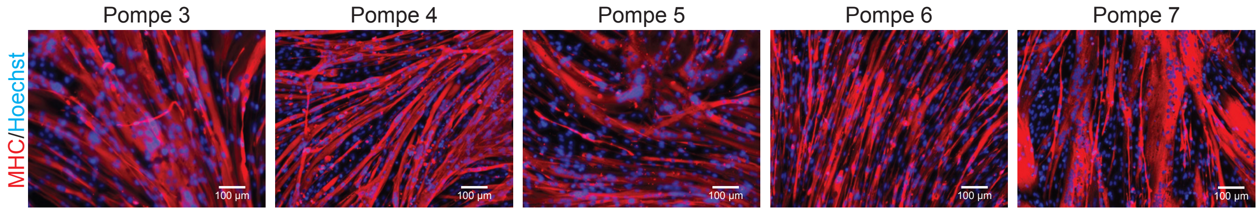

	Pompe 3 average	Pompe 3 sd	Pompe 4 average	Pompe 4 sd	Pompe 5 average	Pompe 5 sd	Pompe 6 average	Pompe 6 sd	Pompe 7 average	Pompe 7 sd
**5-14d**	53,6%	27,0%	54,1%	8,2%	44,8%	10,4%	43,3%	7,8%	33,0%	7,7%
**12-18d**	45,8%	9,7%	42,3%	4,5%	44,3%	7,3%	49,4%	4,1%	52,5%	1,3%
**20-25d**	77,2%	13,7%	49,8%	7,0%	59,0%	19,2%	36,8%	8,3%	53,4%	6,6%
**27-35d**	65,1%	12,9%	37,9%	8,1%	43,6%	2,1%	35,5%	8,7%	38,7%	6,7%

B Control 2

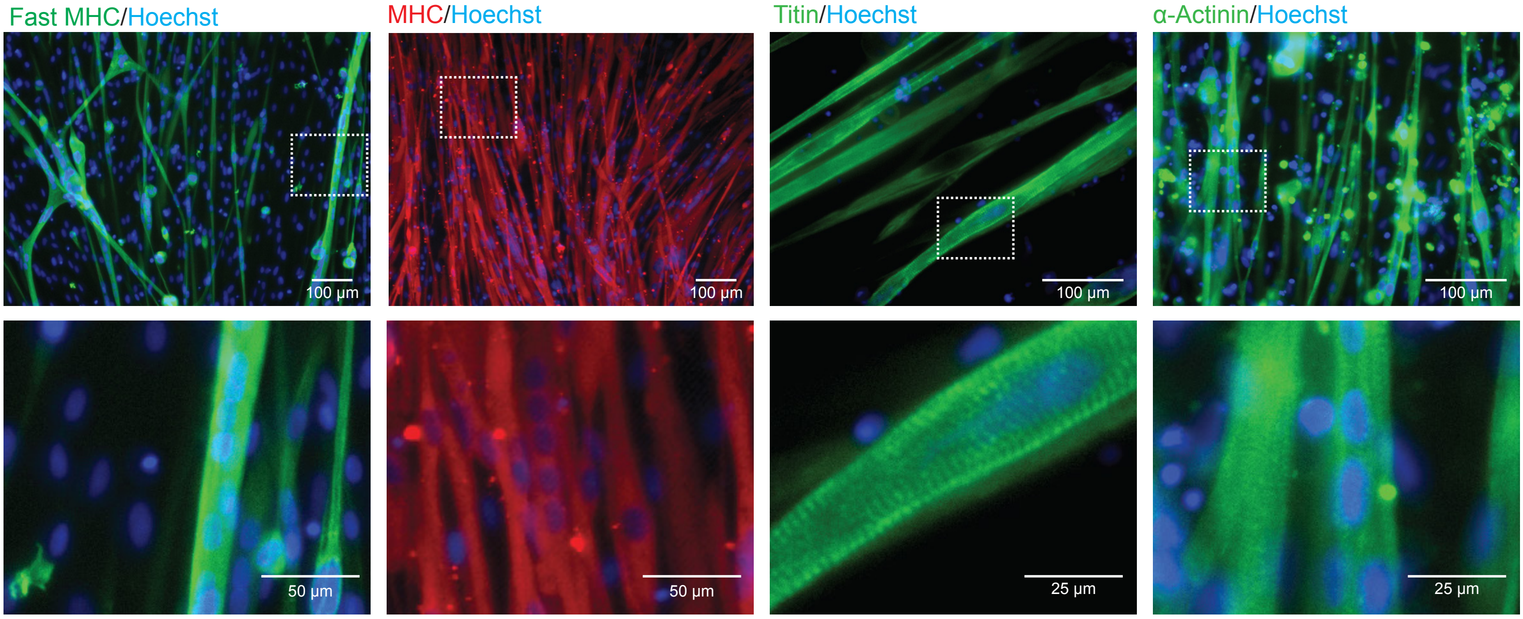

C

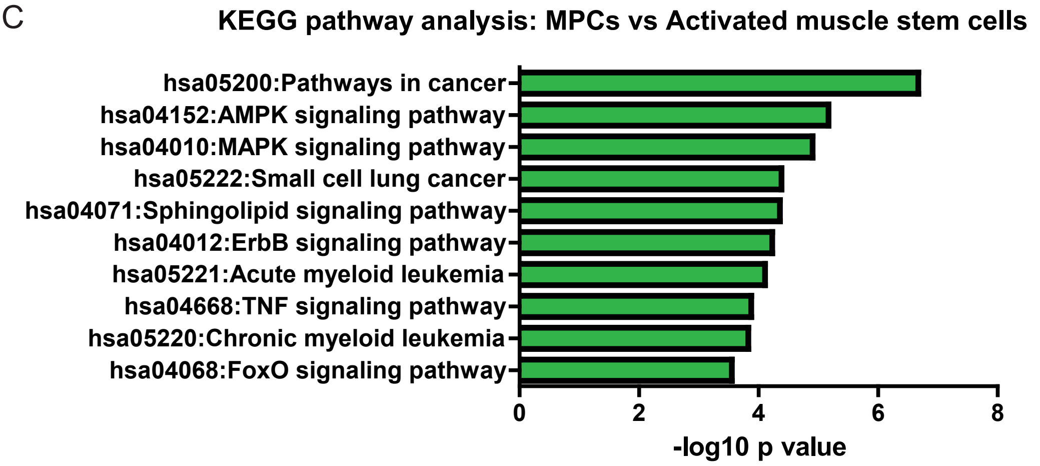

Figure S2

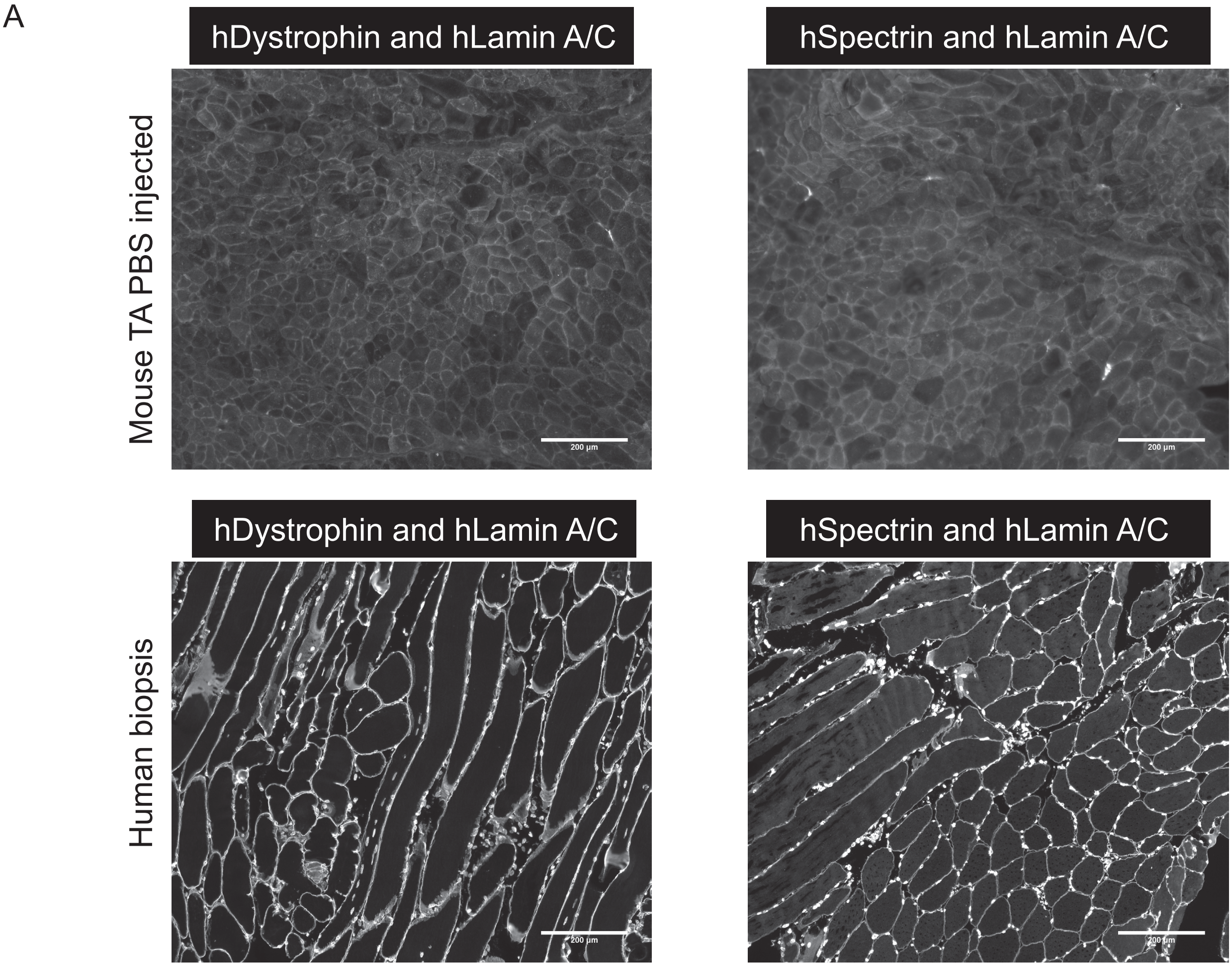
A
hDystrophin and hLamin A/C
hSpectrin and hLamin A/C
Mouse TA PBS injected
hDystrophin and hLamin A/C
hSpectrin and hLamin A/C
Human biopsis

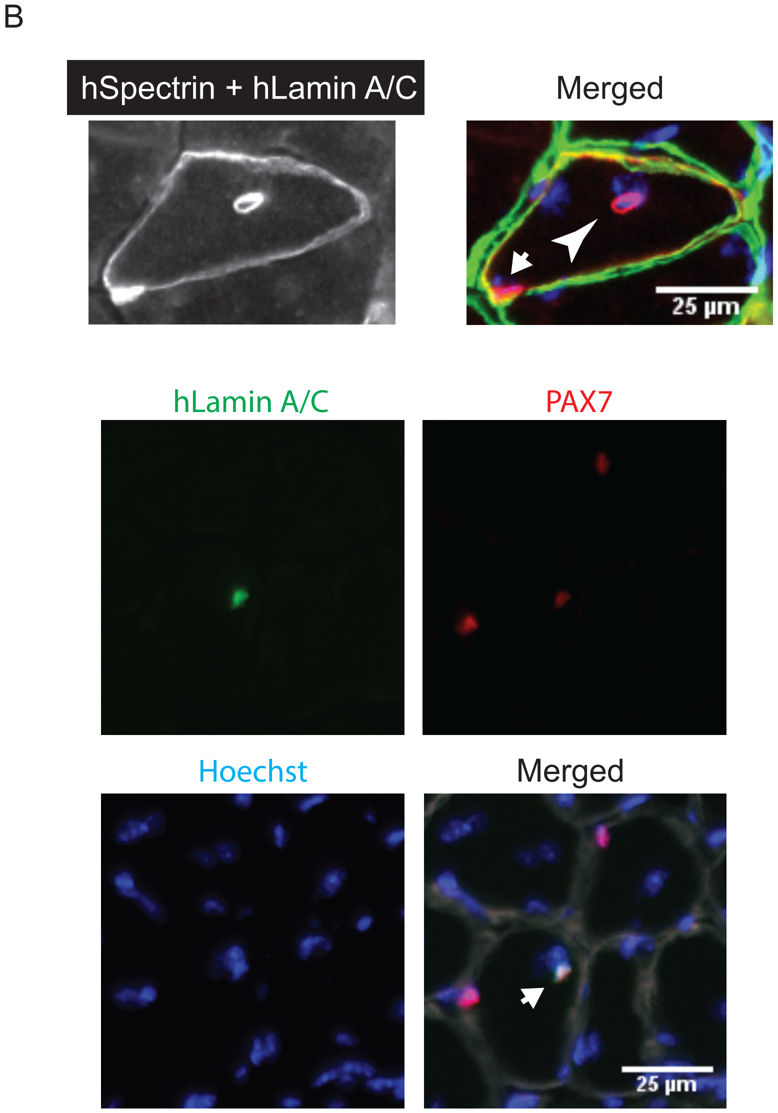
B
hSpectrin + hLamin A/C
Merged
25 μm
hLamin A/C
PAX7
Hoechst
Merged
25 μm

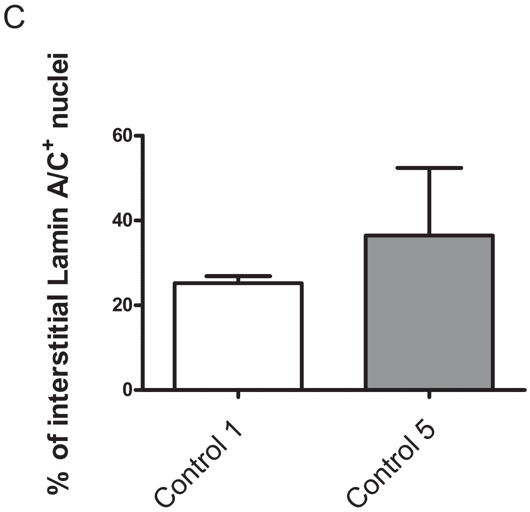
C
% of interstitial Lamin A/C+ nuclei
60
40
20
0
Control 1
Control 5

Figure S3

SUPPLEMENTARY FIGURE LEGENDS

Figure S1 (related to Figure 1): Cell morphologies during differentiation of iPSCs into the myogenic lineage and after purification of myogenic progenitors. (A) Healthy-control iPSCs 3, 4, 5 and 6 were differentiated using a 35-day protocol consisting of GSK3β inhibition, FGF2 treatment, and a minimal medium (see Figure 7). Light microscope (4x magnification) images were taken before and during differentiation at the days indicated. Representative images are shown. (B) One day after FACS purification (described in Figure 7), light microscope images were taken from myogenic progenitors generated from healthy controls 4, 5 and 6 at a magnification of 10x and 20x. Representative images are shown.

Figure S2 (related to Figures 2 and 4): Differentiation, maturation of purified myogenic progenitors, and KEGG pathway analysis. (A) Myosin heavy chain (MHC) staining on 4 days differentiated myogenic progenitors from healthy controls 3- 8 and Pompe 3 – 7. For images of healthy controls 1 and 2, and Pompe 1 and 2, see van der Wal et al., 2017. Nuclei were stained with Hoechst. Images are representative for each differentiation. Fusion index during expansion was quantified and data are mean ± SD of 3 fields per point. (B) Staining of matured fibers from myogenic progenitors of healthy control 2. After 6-8 days of differentiation, cells were stained with Fast MHC, MHC, Titin and α-Actinin antibodies. Nuclei were stained with Hoechst and are shown in blue. (C) KEGG pathway analysis using DAVID of mapped genes comparing myogenic progenitors in proliferation phase (MPCs, this study) versus activated muscle stem cells (Charville et al., 2015). The 10 most significant pathways are shown.

Figure S3 (related to Figure 6): Positive and negative controls for analysis of *in vivo* engraftment and for cell contribution to muscle regeneration *in vivo*. (A) The upper panels show sections from the *tibialis anterior* from immunodeficient mice 4 weeks after injection with PBS only. The lower panel shows sections from a human biopsy of the quadriceps femoris. Sections were analyzed by immunohistochemistry using human specific Lamin A/C, Spectrin and Dystrophin antibodies (white). (B) Upper panel represent two different locations of Lamin A/C^{+} nuclei within the same Spectrin^{+} fiber. The human nuclei on a satellite cell position are indicated with an arrow and the myonuclei with an arrowhead. Lower panel shows a PAX7^{+} (red), Lamin A/C^{+} (green) nucleus in a Laminin^{+} (grey) muscle fiber. (C) Percentage of Lamin A/C^{+} nuclei present at the muscle interstitium per section of each biological replicate. Sections that showed engraftment were used for quantification. Data are mean ± SD (n= 2 TAs transplanted per line used. Each replicate was transplanted in different mice). All sections were counterstained with Hoechst (blue).

SUPPLEMENTARY TABLES

Table S1. Comparison of transgene-free skeletal-muscle differentiation protocols using GSK3β inhibition

	**Purification protocol**	**Fold expansion**	**Cryopreser vation**	**Duration**	**Fusion index**	**Engraftment**
**(Borchin et al., 2013)**	FACS	N.R.	N.R.	35 days	N.R.	N.R.
**(Xu et al., 2013)**	No purification, differentiation analysed in original plate	N.R.	N.R.	36 days	N.R.	Yes, unpurified culture
**(Shelton et al., 2014)**	No purification, differentiation analysed in original plate	N.R.	N.R.	50 days	N.R.	N.R.
**(Chal et al., 2015)**	No purification, differentiation analysed in original plate	N.R.	N.R.	50 days	N.R.	N.R.
**(Shelton et al., 2016)**	No purification, differentiation analysed in original plate	3x	N.R.	50 days	N.R.	N.R.

**(Choi et al., 2016)**	FACS	10^5x	Yes	30 days	10-15%	Yes, unpurified culture
**(Caron et al., 2016)**	Pre-plating	1250x	N.R.	26 days	N.R.	N.R.
**(Chal et al., 2016)**	Pre-plating	N.R.	Yes	35 days	N.R.	N.R.
**(Swartz et al., 2016)**	No purification, differentiation analysed in original plate	N.R.	Yes	36 days	N.R.	N.R.
**(Kim et al., 2017)**	No purification, differentiation analysed in original plate	N.R.	N.R.	50 days	N.R.	Yes, unpurified culture
**(van der Wal et al., 2017)**	FACS	5 x 10^7x	Yes	35 days	60-80%	N.R.
**This study**	FACS	5 x 10^{11}x	Yes	35 days	20-97%	Yes, purified culture

N.R: Not Reported

Table S2. Optimization of CHIR99021 concentration

Control 1

**CHIR99021**	**Days**	**Confluency**	**PAX7$^+$ cells**
3 µM	4	65%	15-10%
3 µM	5	50%	7-10%
3 µM	8	40%	1-2%
3 µM	10	40%	0%
4 µM	4	85%	30-35%
4 µM	5	100%	35-40%
4 µM	8	75%	30-35%
4 µM	10	50%	5-10%
5 µM	4	60%	15-20%
5 µM	5	70%	20-25%
5 µM	8	0%	0%
5 µM	10	0%	0%

Control 2

**CHIR99021**	**Days**	**Confluency**	**PAX7$^+$ cells**
3 µM	4	95%	1-2%
3 µM	5	100%	2-3%
3 µM	8	90%	3-4%
3 µM	10	95%	3-4%
4 µM	4	95%	10-15%
4 µM	5	95%	9-12%
4 µM	8	95%	10-15%
4 µM	10	85%	1-2%
5 µM	4	95%	3-5%
5 µM	5	95%	15-20%
5 µM	8	50%	7-10%
5 µM	10	30%	0%

Table S3. RNA sequencing datasets used in this study

Data source	Accession	Abbreviation	Reference
ENA	ERR975347	Activated Muscle Stem Cell 2	(Charville et al., 2015)
ENA	ERR975349	Activated Muscle Stem Cell 1 P38 treated 2	(Charville et al., 2015)
ENA	ERR975346	Activated Muscle Stem Cell 1	(Charville et al., 2015)
ENA	ERR975348	Activated Muscle Stem Cell 1 P38 treated 1	(Charville et al., 2015)
NCBI	GEO: *GSM3024344*	MPCs control 1A	This study
NCBI	GEO: *GSM3024345*	MPCs control 1B	This study
NCBI	GEO: *GSM3024346*	MPCs control 2A	This study
NCBI	GEO: *GSM3024347*	MPCs control 2B	This study
ENA	ERR975345	Quiescent Muscle Stem Cell 2	(Charville et al., 2015)
ENA	ERR975344	Quiescent Muscle Stem Cell 1	(Charville et al., 2015)
NCBI	GEO: *GSM3024348*	MPCs 4 days differentiated control 1A	This study
NCBI	GEO: *GSM3024349*	MPCs 4 days differentiated control 1B	This study
NCBI	GEO: *GSM3024350*	MPCs 4 days differentiated control 2A	This study
NCBI	GEO: *GSM3024351*	MPCs 4 days differentiated control 2B	This study
NCBI	GEO: *GSM2452280*	Neural stem cell 1	(McGrath et al., 2017)
NCBI	GEO: *GSM2452281*	Neural stem cell 2	(McGrath et al., 2017)
NCBI	GEO: *GSM2452282*	Neural stem cell 3	(McGrath et al., 2017)
ENCODE	ENCBS476ENC	Dermal Fibroblast 1	N/A
ENCODE	ENCBS459ENC	Mesenchymal stem cell 2	N/A
ENCODE	ENCSR828TEI	Primary Myotube 1	N/A
ENCODE	ENCBS018ENC	Chrondocyte 1	N/A
ENCODE	ENCLB014ZZZ	Cardiomyocyte	N/A
ENCODE	ENCBS460ENC	Mesenchymal stem cell 1	N/A
ENCODE	ENCSR000CUI	Myosatellite cell 2	N/A
ENCODE	ENCSR000AAG	Smooth muscle cell	N/A
NCBI	SRX689200	Primary hepatocytes 2	(Kambara et al., 2014)
ENCODE	ENCSR000CUI	Myosatellite cell 1	N/A
ENCODE	ENCBS019ENC	Chrondocyte 2	N/A
ENCODE	ENCBS475ENC	Dermal Fibroblast 2	N/A
ENCODE	ENCBS945YXY	Primary Kidney epithelial cell 2	N/A
NCBI	SRX673854	Primary hepatocytes 1	(Kambara et al., 2014)
ENCODE	ENCBS007YZP	Primary Kidney epithelial cell 1	N/A
ENCODE	ENCSR828TEI	Primary Myotube 2	N/A
ENCODE	ENCSR444WHQ	Primary Myoblast 2	N/A
ENCODE	ENCBS293AAA	Embryonic stem cell 1	N/A

ENCODE	ENCBS624XJG	Embryonic stem cell 2	N/A
ENCODE	ENCSR444WHQ	Primary Myoblast 1	N/A
ENCODE	ENCBS485ENC	Hematopoietic stem cell	N/A

Table S4. Antibodies and primers used in experiments

Name	Dilution or Sequence 5'-3'	Company	Assay
Mouse-anti-MF20	1:50	DSHB	IF
Rabbit-anti-Myogenin	1:100	Santa Cruz (sc-576)	IF
Mouse-anti-PAX7	1:100 or 1:20	DSHB	IF or IHC
Mouse-anti-α-Actinin	1:100	Sigma-Aldrich (A7811)	IF
Mouse-anti-Myosin (fast)	1:100	Sigma-Aldrich (M4276)	IF
Mouse-anti-Titin	1:50	DSHB	IF
Rabbit-anti-Laminin	1:100	Sigma-Aldrich (L9393)	IHC
Mouse-anti-hSpectrin	1:100	Leica (SPEC1-CE)	IHC
Mouse-anti-hDystrophin	1:100	Millipore (MABT827)	IHC
Mouse-anti-hLaminA/C	1:100	Vector Laboratories (VP-L550)	IHC
GAA Exon 1-2 fw	AAACTGAGGCACGGAGCG	IDTDNA	RT-qPCR
GAA Exon 1-2 rv	GAGTGCAGCGGTTGCCAA	IDTDNA	RT-qPCR
Set_1_fw	TTCCCAGGGCCGGTTAATGT	IDTDNA	PCR
Set_1_rv	GCTCTGGGCGGAGGAATATG	IDTDNA	PCR
Set_2_fw	CCTGAGTCCGGACCACTTTG	IDTDNA	PCR
Set_2_rv	CACCGGTTCAATTGCCGAC	IDTDNA	PCR
Set_3_fw	GTCTCTCACTCGGAAGGACAT	IDTDNA	PCR
Set_3_rv	TACCCCGAAGAGTGAGTTTGC	IDTDNA	PCR

SUPPLEMENTARY METHODS

GAA enzyme activity assay

Differentiated myogenic progenitors were harvested with ice-cold protein lysis buffer (50 mM Tris (pH 7.5)), 100 mM NaCl, 50 mM NaF, 1% Triton X-100 and one tablet Protease Inhibitor Cocktail cOmplete, with EDTA, (Roche, Penzberg, Germany) for 10 minutes on ice. GAA enzyme activity was measured as described previously (Kroos et al., 2007). Total protein concentrations were determined with the BCA protein assay kit (Pierce, Thermo Scientific, Waltham, MA).

qRT-PCR

qRT-PCR was measured with a CFX96 real-time system (Bio-Rad, Hercules, CA). cDNA was diluted 5x or 10x times and 4 µL was used in a qRT-PCR reaction consisting of a total volume of 15 µL with 7.5 µL iTaq Universersal SYBR Green Supermix (Bio-Rad, Hercules, CA), 10 pmol/µL forward and reverse primers (Table S4). Per plate, a standard curve was included with 5 dilutions.

Immunofluorescent analysis of *in vitro* differentiation

Myogenic progenitors were stained as described previously (van der Wal et al., 2017). Briefly, cells were permeabilized for 5 minutes with 0.1% Triton X-100 (AppliChem, Darmstadt, Germany) in PBS and blocked for 30 minutes at room temperature in blocking solution (PBS-T (0.1% Tween, Sigma-Aldrich, Irvine, UK) with 3% BSA (Sigma-Aldrich, Irvine, UK)). Primary antibodies (Table S4) were incubated for 1 hour at room temperature and diluted into 0.1% BSA in PBS-T, washed with PBS-T and incubated with secondary antibodies (1:500, Alexa-Fluor-488-α-mouse, A11001, Alexa-Fluor-594-α-rabbit, A10474, Alexa-Fluor-488-α-rabbit,

A11008, Invitrogen, Carlsbad, CA; or horse anti-mouse biotin, BA-2000, Vector Laboratories, Burlingame, CA). When a secondary biotinylated antibody was used, cells were washed three times for 5 minutes with PBS-T and incubated with Streptavidine 594 (1:500, S-32356, Invitrogen, Carlsbad, CA,). The cells were subsequently washed two times for 5 minutes with PBS and incubated for 15 minutes with Hoechst (1:15000, Thermo Scientific, Waltham, MA) before imaging.

Generation of induced pluripotent stem cells

Control iPSC lines were previously reprogrammed, characterized and cultured as described in van der Wal et al. (van der Wal et al., 2017). Healthy control 3 and healthy control 4 iPSCs were a gift from Dr. Mehrnaz and Prof. Joost Gribnau . Healthy control 2 (previously characterized in (Dambrot et al., 2013)), healthy control 5 (LUMC0004iCTRL10), and healthy control 8 (LUMC0030iCTRL12) iPSCs were gifts from Dr. Christian Freund and Prof. Christine Mummery. Using the MycoAlert™ Mycoplasma Detection Kit (Lonza, Walkersville, MD), the iPSC lines were regularly tested for contamination with mycoplasma. All results in this study were obtained with cultures that had tested negative. The identities of cell lines used in this study were confirmed by DNA sequencing.

Generation and expansion of myogenic progenitors from iPSCs

iPSC cultures in 100 mm dishes were used to initiate myogenic differentiation as described previously (van der Wal et al., 2017). Briefly, after 5 days of iPSC expansion, differentiation into myogenic progenitors was started with myogenic differentiation medium (DMEM/F12, 1% Insulin-Transferrin-Selenium-Ethanolamine (ITS-X), 1% penicillin/streptomycin/L-glutamine (P/S/G), all Gibco, Waltham, MA) supplemented with 3.5 μM CHIR99021 (Axon Medchem, Groningen, the Netherlands) for 5 days; and changed to myogenic differentiation medium supplemented with 20 ng/ml FGF2 (Prepotech, Rocky Hill, NJ) for 14 days. For the last 16 days, cells were cultured in myogenic differentiation medium only. Myogenic progenitors were purified using FACS with anti-C-MET-APC (1:50, R&D systems, Minneapolis MN), and anti-HNK-1-FITC (1:100, Aviv Systems Biology, San Diego, CA) antibodies; and Hoechst (33258, Life Technologies, Carlsbad, CA) was added to stain live cells. The c-MET^{+}/Hoechst^{+}/Hnk-1^{-} fraction was sorted in myogenic progenitor proliferation (MMP) medium (DMEM high-glucose supplemented with 10% fetal bovine serum, 1% penicillin/streptomycin/L-glutamine and 100 ng/ml FGF2) supplemented with 1x Revitacell supplement (Gibco, Waltham, MA) on ECM (Sigma-Aldrich, E6909)-coated dishes as described (van der Wal et al., 2017).

SUPPLEMENTARY REFERENCES

Borchin, B., Chen, J., and Barberi, T. (2013). Derivation and FACS-mediated purification of PAX3+/PAX7+ skeletal muscle precursors from human pluripotent stem cells. Stem Cell Reports *1*, 620-631.

Caron, L., Kher, D., Lee, K.L., McKernan, R., Dumevska, B., Hidalgo, A., Li, J., Yang, H., Main, H., Ferri, G., *et al.* (2016). A Human Pluripotent Stem Cell Model of Facioscapulohumeral Muscular Dystrophy-Affected Skeletal Muscles. Stem Cells Transl Med *5*, 1145-1161.

Chal, J., Al Tanoury, Z., Hestin, M., Gobert, B., Aivio, S., Hick, A., Cherrier, T., Nesmith, A.P., Parker, K.K., and Pourquie, O. (2016). Generation of human muscle fibers and satellite-like cells from human pluripotent stem cells in vitro. Nat Protoc *11*, 1833-1850.

Chal, J., Oginuma, M., Al Tanoury, Z., Gobert, B., Sumara, O., Hick, A., Bousson, F., Zidouni, Y., Mursch, C., Moncuquet, P., *et al.* (2015). Differentiation of pluripotent stem cells to muscle fiber to model Duchenne muscular dystrophy. Nat Biotechnol *33*, 962-969.

Charville, G.W., Cheung, T.H., Yoo, B., Santos, P.J., Lee, G.K., Shrager, J.B., and Rando, T.A. (2015). Ex Vivo Expansion and In Vivo Self-Renewal of Human Muscle Stem Cells. Stem Cell Reports *5*, 621-632.

Choi, I.Y., Lim, H., Estrellas, K., Mula, J., Cohen, T.V., Zhang, Y., Donnelly, C.J., Richard, J.P., Kim, Y.J., Kim, H., *et al.* (2016). Concordant but Varied Phenotypes among Duchenne Muscular Dystrophy Patient-Specific Myoblasts Derived using a Human iPSC-Based Model. Cell Rep *15*, 2301-2312.

Dambrot, C., van de Pas, S., van Zijl, L., Brandl, B., Wang, J.W., Schalij, M.J., Hoeben, R.C., Atsma, D.E., Mikkers, H.M., Mummery, C.L., *et al.* (2013). Polycistronic lentivirus induced pluripotent stem cells from skin biopsies after long term storage, blood outgrowth endothelial cells and cells from milk teeth. Differentiation *85*, 101-109.

Kambara, H., Niazi, F., Kostadinova, L., Moonka, D.K., Siegel, C.T., Post, A.B., Carnero, E., Barriocanal, M., Fortes, P., Anthony, D.D., *et al.* (2014). Negative regulation of the interferon response by an interferon-induced long non-coding RNA. Nucleic Acids Res *42*, 10668-10680.

Kim, J., Magli, A., Chan, S.S.K., Oliveira, V.K.P., Wu, J., Darabi, R., Kyba, M., and Perlingeiro, R.C.R. (2017). Expansion and Purification Are Critical for the Therapeutic Application of Pluripotent Stem Cell-Derived Myogenic Progenitors. Stem Cell Reports *9*, 12-22.

Kroos, M.A., Pomponio, R.J., Hagemans, M.L., Keulemans, J.L., Phipps, M., DeRiso, M., Palmer, R.E., Ausems, M.G., Van der Beek, N.A., Van Diggelen, O.P., *et al.* (2007). Broad spectrum of Pompe disease in patients with the same c.-32-13T->G haplotype. Neurology *68*, 110-115.

McGrath, E.L., Rossi, S.L., Gao, J., Widen, S.G., Grant, A.C., Dunn, T.J., Azar, S.R., Roundy, C.M., Xiong, Y., Prusak, D.J., *et al.* (2017). Differential Responses of Human Fetal Brain Neural Stem Cells to Zika Virus Infection. Stem Cell Reports *8*, 715-727.

Shelton, M., Kocharyan, A., Liu, J., Skerjanc, I.S., and Stanford, W.L. (2016). Robust generation and expansion of skeletal muscle progenitors and myocytes from human pluripotent stem cells. Methods *101*, 73-84.

Shelton, M., Metz, J., Liu, J., Carpenedo, R.L., Demers, S.P., Stanford, W.L., and Skerjanc, I.S. (2014). Derivation and expansion of PAX7-positive muscle progenitors from human and mouse embryonic stem cells. Stem Cell Reports *3*, 516-529.

Swartz, E.W., Baek, J., Pribadi, M., Wojta, K.J., Almeida, S., Karydas, A., Gao, F.B., Miller, B.L., and Coppola, G. (2016). A Novel Protocol for Directed Differentiation of C9orf72-Associated Human Induced Pluripotent Stem Cells Into Contractile Skeletal Myotubes. Stem Cells Transl Med *5*, 1461-1472.

van der Wal, E., Bergsma, A.J., van Gestel, T.J.M., In 't Groen, S.L.M., Zaehres, H., Arauzo-Bravo, M.J., Scholer, H.R., van der Ploeg, A.T., and Pijnappel, W. (2017). GAA Deficiency in Pompe Disease Is Alleviated by Exon Inclusion in iPSC-Derived Skeletal Muscle Cells. Mol Ther Nucleic Acids *7*, 101-115.

Xu, C., Tabebordbar, M., Iovino, S., Ciarlo, C., Liu, J., Castiglioni, A., Price, E., Liu, M., Barton, E.R., Kahn, C.R., *et al.* (2013). A zebrafish embryo culture system defines factors that promote vertebrate myogenesis across species. Cell *155*, 909-921.
