# Supplementary material for: Large-Scale Expansion of Human iPSC-Derived Skeletal Muscle Cells for Disease Modeling and Cell-Based Therapeutic Strategies
Source: Stem Cell Reports. 2018 May 3;10(6):1975–90. doi: 10.1016/j.stemcr.2018.04.002 (PMC5993675; doi:10.1016/j.stemcr.2018.04.002)
Supplement: Document S2. Article plus Supplemental Information [file mmc5.pdf]

Stem Cell Reports
Resource
OPEN ACCESS

Large-Scale Expansion of Human iPSC-Derived Skeletal Muscle Cells for Disease Modeling and Cell-Based Therapeutic Strategies

Erik van der Wal,[1,2,3] Pablo Herrero-Hernandez,[1,2,3] Raymond Wan,[4] Mike Broeders,[1,2,3] Stijn L.M. in 't Groen,[1,2,3] Tom J.M. van Gestel,[1,2,3] Wilfred F.J. van IJcken,[5] Tom H. Cheung,[4] Ans T. van der Ploeg,[2,3] Gerben J. Schaaf,[1,2,3] and W.W.M. Pim Pijnappel[1,2,3,*]
[1]Department of Clinical Genetics, Erasmus University Medical Center, 3015 GE Rotterdam, Netherlands
[2]Department of Pediatrics, Erasmus University Medical Center, 3015 GE Rotterdam, Netherlands
[3]Center for Lysosomal and Metabolic Diseases, Erasmus University Medical Center, 3015 GE Rotterdam, Netherlands
[4]Division of Life Science, Center for Stem Cell Research, Center of Systems Biology and Human Health, State Key Laboratory in Molecular Neuroscience, Hong Kong University of Science & Technology, Clear Water Bay, Kowloon, Hong Kong 999077, China
[5]Erasmus Center for Biomics, Erasmus University Medical Center, 3000 CA Rotterdam, Netherlands
*Correspondence: w.pijnappel@erasmusmc.nl
https://doi.org/10.1016/j.stemcr.2018.04.002

SUMMARY

Although skeletal muscle cells can be generated from human induced pluripotent stem cells (iPSCs), transgene-free protocols include only limited options for their purification and expansion. In this study, we found that fluorescence-activated cell sorting-purified myogenic progenitors generated from healthy controls and Pompe disease iPSCs can be robustly expanded as much as 5×10^{11}-fold. At all steps during expansion, cells could be cryopreserved or differentiated into myotubes with a high fusion index. *In vitro*, cells were amenable to maturation into striated and contractile myofibers. Insertion of *acid α-glucosidase* cDNA into the *AAVS1* locus in iPSCs using CRISPR/Cas9 prevented glycogen accumulation in myotubes generated from a patient with classic infantile Pompe disease. *In vivo*, the expression of human-specific nuclear and sarcolemmar antigens indicated that myogenic progenitors engraft into murine muscle to form human myofibers. This protocol is useful for modeling of skeletal muscle disorders and for using patient-derived, gene-corrected cells to develop cell-based strategies.

INTRODUCTION

Although over 700 human genetic disorders are known that affect skeletal muscle (Kaplan and Hamroun, 2015), very few therapies are available. Skeletal muscle nonetheless has a high capacity for regeneration after injury (Baghdadi and Tajbakhsh, 2017; Bursac et al., 2015; Dumont et al., 2015). Muscle regeneration is mediated by satellite cells (SCs) (Lepper et al., 2011; Murphy et al., 2011; Sambasivan et al., 2011); i.e., adult stem cells located between the sarcolemma and the plasma membrane (Mauro, 1961) that are quiescent in healthy, uninjured muscle. Upon injury, SCs expand to contribute to fiber formation and to self-renew the SC pool.

SCs are considered useful for *in vitro* disease modeling to investigate molecular mechanisms of disease, test drugs, or develop cell-based therapies. To decipher molecular mechanisms of disease, it is important to generate isogenic controls, given the high variability of gene expression and functional parameters between individuals (Hockemeyer and Jaenisch, 2016; Soldner et al., 2011). To develop cell-based therapy, the ultimate goal is to engraft gene-corrected, autologous cells. However, it has not proved easy to date to establish robust *in vitro* disease models for skeletal muscle disorders, to efficiently restore gene function in skeletal muscle cells, and to develop cell-based therapeutic strategies based on muscle regeneration.

Pluripotent stem cells (PSCs) offer a potential source of skeletal muscle cells. PSCs, including induced PSCs (iPSCs), are easily expanded and maintain their full stem cell potential (Takahashi and Yamanaka, 2016). Differentiation of PSCs to SC-like cells was difficult until the recent development of two major strategies, the first involving the inducible overexpression of PAX7, the master transcription factor for SCs (Darabi et al., 2012). After generation from human embryonic stem cells and iPSCs, purified SC-like cells showed capacity for *in vitro* expansion and differentiation, and also for *in vivo* engraftment and contribution to muscle-fiber formation in immunodeficient mice (Darabi et al., 2012; Magli et al., 2017). The second strategy involved the use of small molecules to develop transgene-free differentiation. After using GSK3β inhibition to activate the Wnt pathway, the basic procedure consists of treatment with fibroblast growth factor 2 (FGF2) and culturing in a minimal medium (see Table S1) (Borchin et al., 2013; Caron et al., 2016; Shelton et al., 2014, 2016; van der Wal et al., 2017b; Xu et al., 2013). In some cases, differentiation into the myogenic lineage has been promoted by including BMP4 inhibition (Chal et al., 2015, 2016; Swartz et al., 2016). In others, FGF2 has been replaced by the Notch signaling inhibitor DAPT (Choi et al., 2016).

Transgene-free protocols can be divided into those that use fluorescence-activated cell sorting (FACS) purification (Borchin et al., 2013; Choi et al., 2016; van der Wal et al.,

This is an open access article under the CC BY license (http://creativecommons.org/licenses/by/4.0/).

2017b) and those that use unpurified cell mixtures or partial purification through preplating (Caron et al., 2016; Chal et al., 2015; Shelton et al., 2014; Swartz et al., 2016; Xu et al., 2013) (Table S1). Upon terminal differentiation *in vitro*, unpurified/partially purified myogenic progenitors showed matured myotubes and even myofibers (Chal et al., 2015, 2016; Swartz et al., 2016). Three reports showed engraftment of myogenic cells from unpurified cultures into immunodeficient mice (Choi et al., 2016; Kim et al., 2017; Xu et al., 2013). Choi et al. (2016) reported that purification of myogenic progenitors by FACS resulted in myogenic progenitors that could be expanded 10^5-fold. Upon *in vitro* differentiation to myotubes, these cells also showed a low (10%–15%) fusion index (Table S1).

In vivo engraftment of purified myogenic progenitors using a transgene-free procedure has not been reported so far. Similarly, it has not been possible yet to expand transgene-free, purified myogenic progenitors and differentiate and mature these cells to myotubes with high fusion index. Recently, we have modified a protocol by Borchin et al. (2013) for the transgene-free differentiation of human iPSC into SC-like cells, and used a simplified FACS purification procedure that selects C-MET-expressing cells that are HNK negative (Borchin et al., 2013; van der Wal et al., 2017b). The purified cells could be expanded at least 5×10^7-fold and cryopreserved. At any point during the expansion, cells could be differentiated into myotubes with a high (60%–80%) fusion index. We have applied this protocol to model Pompe disease, which is a progressive inheritable metabolic myopathy caused by deficiency of acid α-glucosidase (*GAA*), resulting in lysosomal glycogen accumulation (van der Ploeg and Reuser, 2008). This protocol allowed the quantitative analysis of the effects of antisense oligonucleotides designed to restore canonical pre-mRNA splicing of *GAA* in skeletal muscle cells from Pompe patients (van der Wal et al., 2017a).

Here, we further explored the expansion capacity and the *in vitro* and *in vivo* potential of myogenic progenitors, generated from iPSCs in a transgene-free manner and FACS purified, for the future development of therapies for skeletal muscle disorders.

RESULTS

Optimization of the Generation of Myogenic Progenitors from iPSCs

As a starting point, we took the protocol published by Borchin et al. (2013), which we had modified recently (van der Wal et al., 2017b). This protocol consists of treating human iPSCs first with the GSK3β inhibitor CHIR99021, then with FGF2, followed by prolonged culturing in minimal medium. The treatment with CHIR99021 is a critical step, as too-low concentrations fail to yield myogenic progenitors, while too-high concentrations can be toxic. The optimal concentration most likely depends on the cell culture conditions used. We assume, for example, that the outcome can be affected by culturing iPSCs with or without feeders.

In our experiments, we cultured iPSCs on γ-irradiated mouse embryonic fibroblasts. To determine the optimal treatment with CHIR99021, we varied the concentration and duration of treatment and scored for confluency and PAX7 expression (Table S2). The results in two independent iPSC lines showed that the highest number of PAX7$^+$ cells was induced after 4–5 days at a concentration of 4 μM CHIR99021 in the absence of toxicity. To avoid any risk of toxicity in subsequent experiments, we chose 5-day incubation at a concentration of 3.5 μM CHIR99021.

Robustness of the Myogenic Differentiation Protocol

As outlined in Figure 1A, we used primary fibroblast-derived iPSCs from 15 different donors, applying the myogenic differentiation procedure in over 50 individual differentiation experiments. Eight of these iPSC lines were derived from healthy individuals, while seven were from patients with Pompe disease. Figure 1B shows robust generation of PAX7$^+$ areas in six examples of healthy control iPSCs after 35 days of differentiation as described previously (van der Wal et al., 2017b). During the differentiation procedure, phase-contrast microscopy showed small colonies with a confluency of between 20% and 40% at day 1 (Figure S1A). After 5 days of culture, iPSC colonies had reached a medium size. At this stage CHIR99021 treatment was started. After 5 days of incubation, we observed increased cell detachment, which was attenuated after a further 3–4 days in FGF2-containing medium. From day 17 onwards, the cells started to proliferate rapidly, and cultures reached complete confluency after 24 days. Multinucleated myotube-like cells were observed between 30 and 40 days. During this differentiation procedure, we observed similar morphological changes in all iPSC lines (Figure S1A and data not shown).

Differentiation of 59 cultures from a total of 15 donors yielded an average of 4.26% ± 3.96% of C-MET$^+$/Hoechst$^+$/HNK-1$^-$ cells (Figure 1C). There were no significant differences in the number of C-MET$^+$/Hoechst$^+$ cells between iPSCs from healthy controls and from Pompe patients. Sorting differentiation cultures with low levels of C-MET$^+$/Hoechst$^+$/HNK-1$^-$ cells (~0.2% of cells) resulted in expandable myogenic progenitors whose differentiation capacity was similar to that of cultures with a high recovery (>2%) (data not shown). C-MET-/HNK-1$^+$ cells were unable to form myosin heavy chain (MHC)-positive cells after 4 days of differentiation (data not shown). After 24 hr of plating, sorted myogenic progenitors revealed a rather

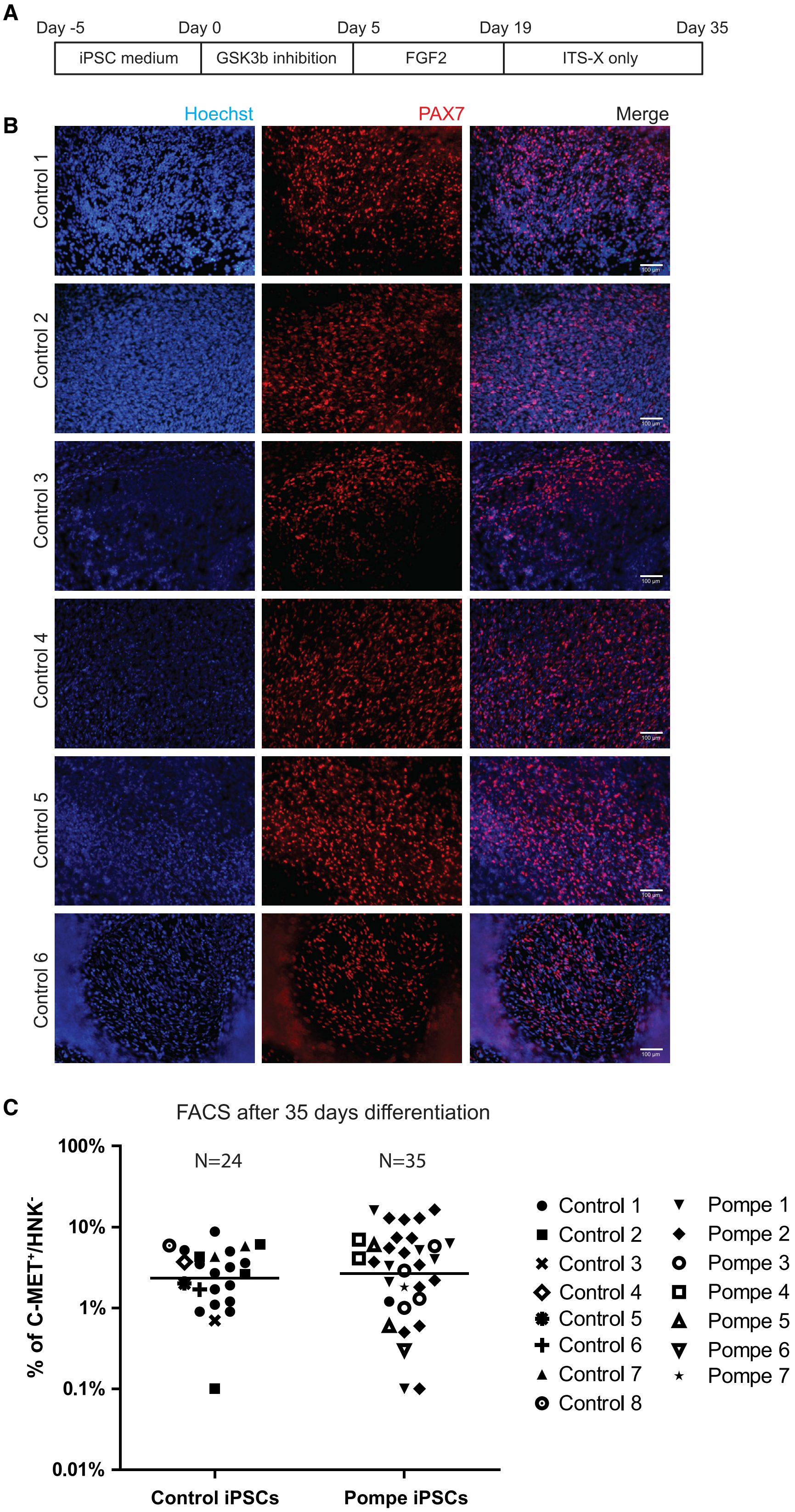

Figure 1. Robustness of Generation and Purification of Myogenic Progenitors from iPSCs

(A) Scheme for myogenic differentiation of iPSCs.

(B) Representative examples of PAX7$^+$ cells obtained in the original culture dishes from six different control iPSC lines that were differentiated using a 35-day protocol consisting of consecutive treatment with CHIR, FGF2, and minimal medium (Borchin et al., 2013; van der Wal et al., 2017b). The other two control iPSC lines showed similar patches of PAX7$^+$ cells (data not shown). Red: PAX7$^+$ nuclei using immunofluorescent staining. Blue: nuclei stained with Hoechst. For images of the plates during this 35-day protocol see Figure S1A.

(C) Differentiations described in (B) were purified using a one-step FACS purification based on selection for C-MET$^+$ myogenic cells and counter selection of HNK1$^+$ neural crest cells (Borchin et al., 2013). Results are shown for 59 differentiations performed on iPSCs derived from eight healthy controls and seven Pompe patients. Each symbol represents an individual differentiation experiment. Means are indicated by horizontal lines. A total number of 24 differentiations of control iPSCs and 35 differentiations of Pompe iPSCs were performed.

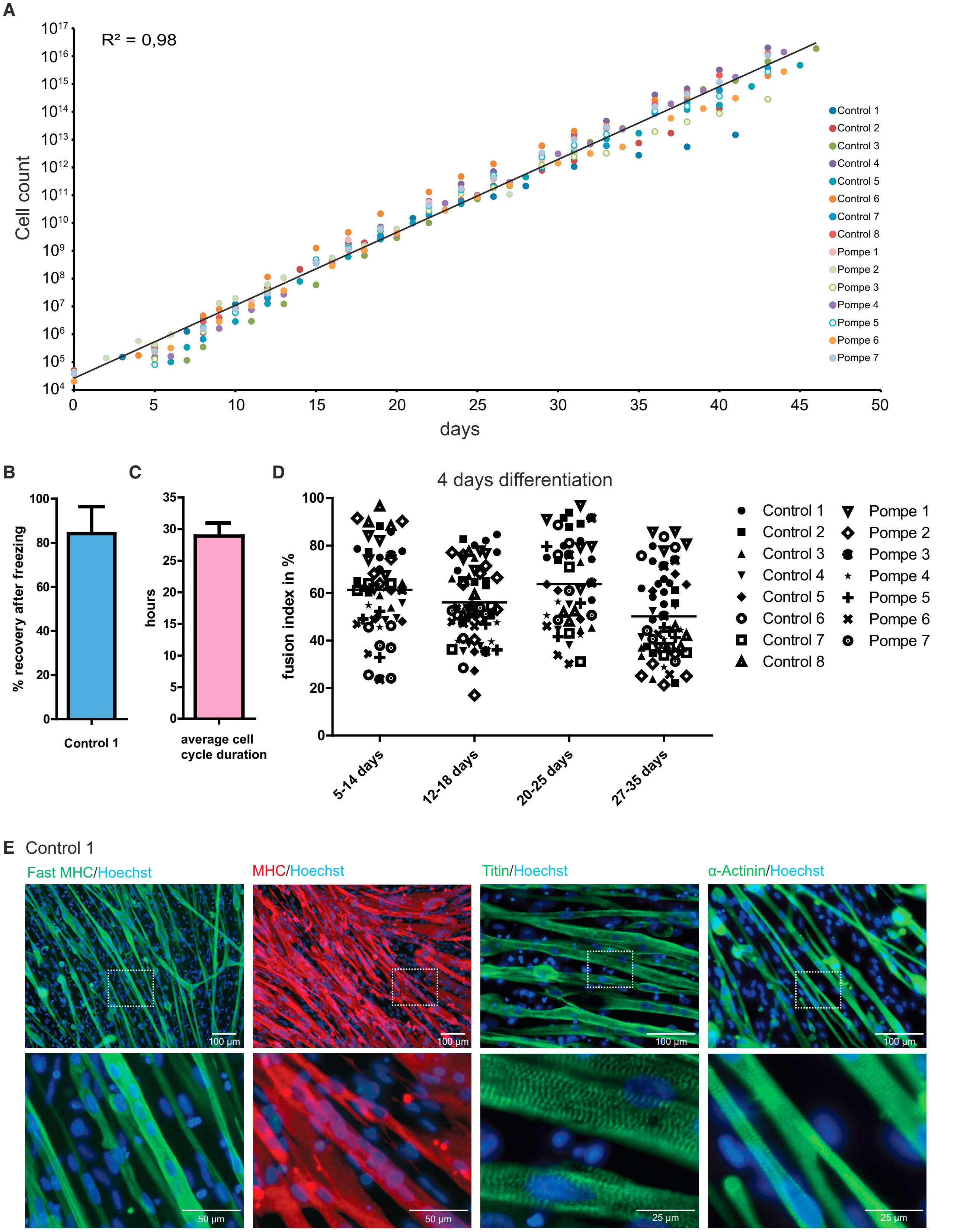

(legend on next page)

uniform morphology (Figure S1B). These results demonstrated that the differentiation protocol robustly generated C-MET$^+$/Hoechst$^+$/HNK$^-$ myogenic cells.

In Vitro Expansion, Differentiation, and Maturation of Purified Myogenic Progenitors

During 31 days of culture we had previously determined the proliferation rate of purified myogenic progenitors derived from two healthy controls and two Pompe patients (van der Wal et al., 2017b). To further determine expansion capacity, we determined the expansion capacities of myogenic progenitors generated from iPSCs from six additional healthy controls and five additional patients with Pompe disease. Proliferation rates were observed for all myogenic progenitor lines that reached 2×10^{16} cells during 43 days of expansion (Figure 2A). Cells could be cryopreserved with 84% recovery (Figure 2B) without affecting differentiation capacity (data not shown), and showed an average cell cycle of 28.9 hr (Figure 2C). After 43 days of expansion, the proliferation rate diminished, the morphology of cells changed, and differentiation capacity decreased (data not shown). This showed that the myogenic progenitors generated with this protocol could be expanded by a maximum of 5×10^{11}-fold.

Previously we had used a 4-day differentiation protocol to demonstrate that the differentiation capacity remained intact during the expansion phase of myogenic progenitors, based on similar fusion indexes (van der Wal et al., 2017b). Here we extended this analysis to demonstrate that all myogenic progenitors derived from eight healthy control and seven Pompe iPSCs retain their capacity to differentiate into multinucleated myotubes during expansion (Figure 2D). The average fusion index ranged between 20% and 97% and showed no expansion-induced differences (Figure S2A). Next, we tested whether maturation to contractile skeletal muscle cells is possible from purified myogenic progenitors. However, extending culture of myogenic progenitor-derived myotubes in conventional differentiation medium (1% ITS-X [insulin-transferrin-selenium-ethanolamine] in DMEM/F12) beyond day 4 of differentiation increased cell detachment and death (data not shown). Supplementation of the myogenic progenitors' differentiation medium with 0.5%–2% fetal bovine serum increased the overall survival of the culture but also increased the proliferation rate of mononucleated cells, resulting in overgrowth of the cell culture (data not shown). In contrast, supplementation with 1% knockout serum replacement supported further differentiation of myogenic progenitors into skeletal muscle cells for up to 12 days. Longer differentiation resulted in fibers that expressed fast MHC, MHC, titin, and α-actinin; that showed patterns of striation (Figures 2E and S2B); and that contracted spontaneously (Videos S1 and S2). This demonstrated that functional sarcomeres, the strongest evidence of terminal differentiation, were formed.

Generation of Gene-Corrected Myogenic Progenitors Using CRISPR/Cas9-Mediated Insertion of a cDNA into a Safe Harbor

Using gene editing, it is possible to perform genetic correction of human disease *in vitro* by placing an extra copy of the wild-type gene into a so-called safe harbor; i.e., a safe location of the genome (Hockemeyer and Jaenisch, 2016). As such a strategy relies on homology-directed DNA repair, which is inefficient, we generated a targeting construct that allows the selection and subsequent removal of the selection marker. The generic donor vector is shown in Figure 3A. As a proof of concept, we chose the PPP1R12C gene in the AAVS1 locus (Figure 3B) (Lombardo et al., 2011). As well as unique restriction sites that enable cloning of the 5′ and 3′ homology arms, the donor vector contains a ubiquitous EF1α promoter in front of the cDNA of interest (flanked by unique restriction sites); a poly(A) site; and a *neomycin* selection marker driven by the CAG promoter flanked by loxP sites, which provide the option of removing the selection marker by transient expression of CRE recombinase (Figure 3A).

As proof of principle, we aimed to correct the glycogen accumulation caused by deficiency of lysosomal acid alpha glucosidase (GAA) in skeletal muscle cells of Pompe patients *in vitro*. To this end, we cloned the native *GAA* cDNA in the donor construct. iPSCs were generated from

Figure 2. *In Vitro* Expansion, Differentiation, and Maturation of Purified Myogenic Progenitor Cells

(A) Proliferation curves of myogenic progenitors derived from 15 iPSC lines derived from healthy controls or Pompe patients, cultured in proliferation medium. An exponential trend line was plotted and an R^2 was calculated from all data points, which showed similar proliferation rates for all cell lines.

(B) Recovery of control 1 myogenic progenitors from freezing. Data are means ± SD from three independent cultures.

(C) Average cell cycle duration of all cell lines shown in (A). Data are means ± SD of all cell lines shown in (A).

(D) After expansion for the number of days indicated on the X axis, skeletal muscle differentiation was induced for 4 days by switching to differentiation medium. The fusion index was quantified after staining for MHC and Hoechst. Individual values of random fields per cell line (n = 3–5 fields per cell line) are plotted as symbols. Mean values of all cell lines per expansion period are indicated as horizontal lines.

(E) At day 8 of differentiation, myotubes further matured as indicated by staining for fast MHC, MHC, titin, and α-actinin, a striated pattern, and spontaneous contractions (see Videos S1 and S2). Blue, nuclei as stained with Hoechst.

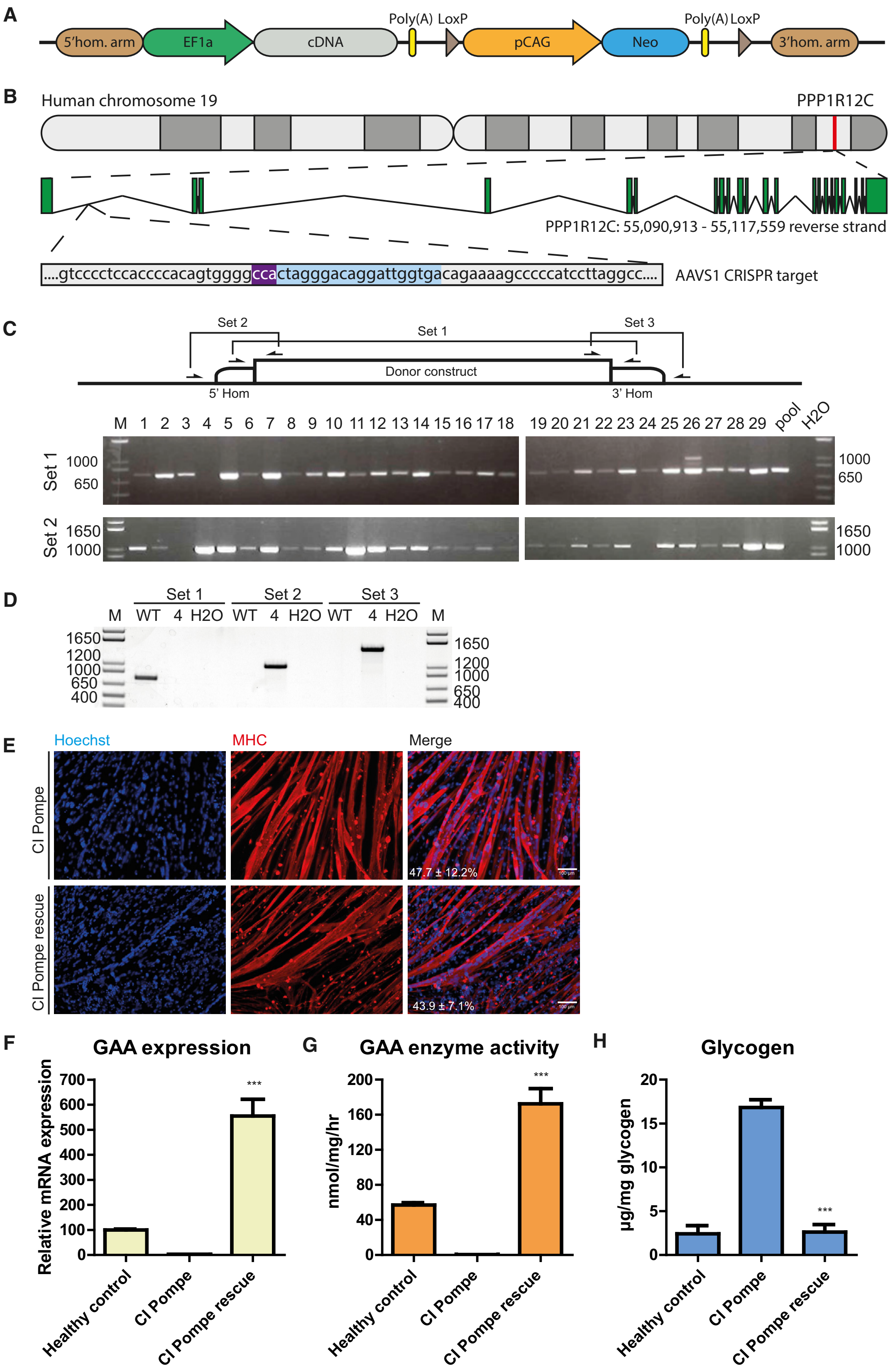

(legend on next page)

a patient with classic infantile (CI) Pompe disease (the most severe phenotype, which is characterized by complete deficiency of GAA enzyme activity), and co-transfected the donor vector containing the *GAA* cDNA with vectors that expressed a guide RNA targeting the AAVS1 locus and a human codon-optimized Cas9 nuclease. After selection with G418, an average of 200 colonies were obtained per 2×10^6 cells, suggesting a targeting frequency of 1×10^{-4}%. Twenty-nine colonies were picked and genotyped using two PCR strategies (Figure 3C). With PCR primer set 1, the untargeted allele yields a product of 749 bp, while the targeted allele yields a product that is too large to be amplified under the conditions employed. With primer set 2, insertion of the *GAA* cDNA at the correct location is detected. The results with primer set 2 showed that 27/29 colonies had inserted the *GAA* cDNA at the desired location. With primer set 1, 28/29 colonies showed that the second allele had not been targeted. One colony (clone 4) contained two targeted alleles. iPSCs from clone 4 were expanded, and the correct integration site was further validated at the 3′ site using primer set 3 (Figure 3D). iPSCs from clone 4 were expanded, and myogenic progenitors were generated and compared with myogenic progenitors from the original iPSC line before gene editing. Myogenic progenitors from these lines were purified, expanded, and subjected to myotube differentiation. Similar differentiation capacities and fusion indexes were observed before and after gene editing (Figure 3E). RT-qPCR analysis showed the absence of *GAA* mRNA expression in the untargeted Pompe myotubes; this was caused by mRNA decay following a frameshift in both alleles (*GAA* genotype c.525del/c.525del). In the gene-edited myotubes, *GAA* mRNA expression had been restored 5.5-fold over levels in healthy control myotubes (Figure 3F). GAA enzyme activity measurements showed complete restoration of GAA activity in the gene-edited myotubes to levels that were ~3-fold higher than those of healthy control myotubes (Figure 3G). Myotubes from the CI Pompe patient showed accumulation of glycogen that was restored in the gene-edited myotubes to the levels of healthy control myotubes (Figure 3H). Altogether, these results demonstrate the feasibility of combining gene editing in iPSCs with the myogenic differentiation protocol to generate gene-corrected skeletal muscle cells.

Expression Profiling of iPSC-Derived Myogenic Progenitors

To characterize myogenic progenitors, we used RNA sequencing (RNA-seq) to perform genome-wide mRNA expression analysis. Profiles from purified, expanded (~15 days) iPSC-derived myogenic progenitors from healthy controls were compared with publicly available datasets (see Table S3) on cell types of different lineages, including adult SCs (FACS purified), myoblasts/myosatellite cells (prepared using preplating), neuronal cells, chondrocytes, cardiomyocytes, hepatocytes, embryonic stem cells, smooth-muscle cells, mesenchymal stem cells, and fibroblasts (Figure 4A). The "new Tuxedo" pipeline (Pertea et al., 2016) was used. Spearman correlation analysis showed that profiles of two independent biological replicates of myogenic progenitors from independent individuals clustered together, indicating that these cells contained similar and defined gene expression profiles (Figure 4, myogenic progenitors from the present study are indicated in green). The profiles of myogenic progenitors clustered away from all other cell types, while the profiles of the adult quiescent and activated muscle stem cells showed an early split from all other profiles. A total of 1,852 out of 13,193 genes were differentially expressed between activated muscle stem cells and myogenic progenitors (false discovery rate < 0.01; Table S5). The dissimilarity between quiescent and

Figure 3. Gene Editing in iPSCs Restores the Pompe Disease Phenotype in Skeletal Muscle Cells *In Vitro*

(A) Generic construct for insertion of a cDNA in a safe harbor following CRISPR/Cas9-mediated targeting.

(B) The construct shown in (A) was tailored to express *GAA* in the AAVS1 locus. After transfection into iPSCs from a classic infantile (CI) Pompe patient, G418 selection was used, and single colonies were picked.

(C) Genotyping was performed using PCR. Primer sets 2 and 3 amplified a product that is only present in correctly targeted clones, while primer set 1 spanned the insertion site to give a product only in the absence of targeting. With primer set 1, 28/29 clones were positive, indicating that most clones also contained an untargeted allele; with primer set 2, 27/29 clones were positive, indicating that most clones showed efficient targeting of at least one allele.

(D and E) (D) One clone (#4) showed targeting of both alleles, which was validated using primer set 3, and was differentiated into myogenic progenitors for further analysis. Myogenic progenitors were generated from healthy controls, a CI Pompe patient (CI Pompe), and the isogenic, gene-corrected, CI Pompe patient (CI Pompe rescue). Myogenic progenitors were purified, expanded, differentiated for 6 days into myotubes, and the fusion index was determined (E).

(F–H) Myogenic progenitors were analyzed for *GAA* mRNA expression at day 4 (F); GAA enzyme activity at day 4 (G); and glycogen accumulation at day 6 (H). *GAA* mRNA expression was measured by RT-qPCR using primers spanning exon 1–2. GAA enzyme activity was measured using the 4-methylumbelliferone assay. Glycogen accumulation was measured biochemically. To deplete cytoplasmic glycogen, cells were cultured in glucose-free medium for the last 24 hr, as described in Bergsma et al. (2015). For (F, G, and H), data are means ± SD of two independent (healthy control) or three independent (CI Pompe disease and rescue) cultures. Two-tailed Student's t test: ***$p < 0.001$.

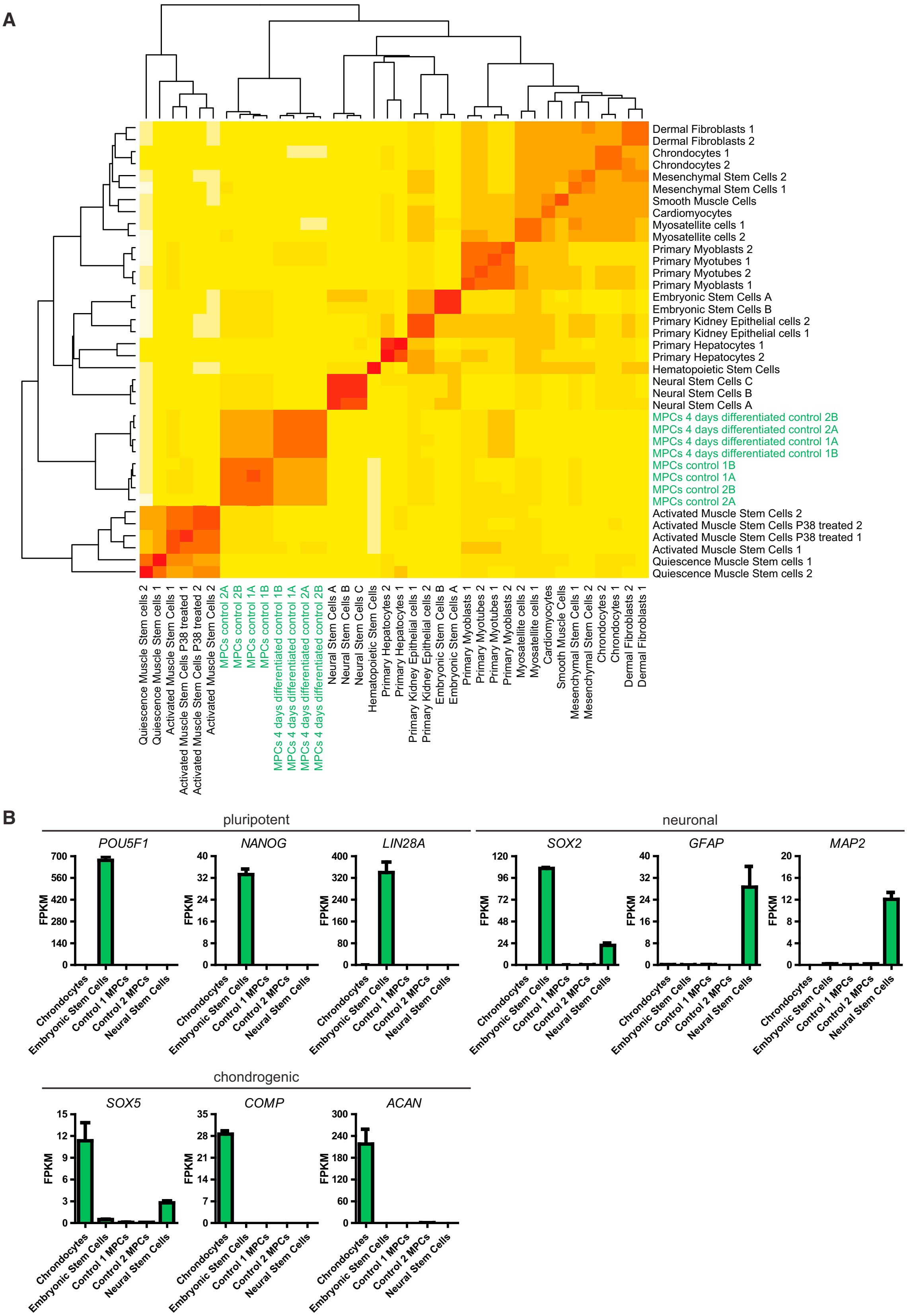

(legend on next page)

activated muscle stem cells from myosatellite cells and primary myoblasts can be explained by the fact that the former cells were FACS purified, while the latter cells were obtained using preplating and probably contained contaminating cell types. KEGG (Kyoto Encyclopedia of Genes and Genomes) pathway analysis of genes that were differentially expressed in myogenic progenitors relative to activated muscle stem cells showed enrichment of the AMPK, MAPK, and ErbB signaling pathways in myogenic progenitors (Figure S2C). These pathways have been involved in cell cycle regulation, muscle regeneration, and/or satellite cell function (Charville et al., 2015; Golding et al., 2007; Theret et al., 2017). Overall, this suggests that the myogenic progenitors were dissimilar from the other cell types tested and contained a defined mRNA expression profile.

To assess the purity of the myogenic progenitors, we used the datasets shown in Figure 4A to examine the expression of markers for pluripotent cells (*POU5F1*, *NANOG*, and *LIN28A*), neuronal cells (*SOX2*, *GFAP*, and *MAP2*), and chondrogenic cells (*SOX5*, *COMP*, and *ACAN*). None of these markers were expressed in the purified iPSC-derived myogenic progenitor cultures, suggesting that contaminating cells from the lineages tested were absent (Figure 4B).

In earlier work we showed that, upon expansion, purified iPSC-derived myogenic progenitors express several myogenic markers, including the MyoD protein (van der Wal et al., 2017b). To examine PAX7 protein expression during *in vitro* expansion and differentiation, we used a PAX7 antibody to perform immunofluorescent analysis. Under proliferating conditions, expanded myogenic progenitors (~25 days) from two independent iPSCs expressed PAX7 in a subset of cells (Figure 5A). Although myogenic progenitor cultures contained a stable ~3% of $PAX7^+$ cells during the majority of the expansion period, the percentage of $Pax7^+$ cells started to decline at day 39 (control 1) or day 28 (control 2) (Figure 5B). After differentiation to myotubes, $PAX7^+$ cells remained present in the culture (Figure 5C and data not shown). These results indicate that, during expansion, a subset of iPSC-derived myogenic progenitors continue to express markers of SCs during both proliferation and differentiation.

In Vivo Myogenic Potential of Myogenic Progenitors

To test the capacity of purified and expanded myogenic progenitors to engraft and contribute to muscle regeneration *in vivo*, we performed cell transplantations in tibialis anterior (TA) muscles of NSG immunodeficient recipient mice that had been pre-injured with $BaCl_2$. Analysis of engraftment was performed 4 weeks after transplantation. Using human-specific epitopes (Lamin A/C, Spectrin, and Dystrophin; for controls, see Figure S3A), we observed that myogenic progenitors that had been expanded for 3 days were able to engraft and participate in the formation of new myofibers (Figure 6A). In addition, myogenic progenitors were engrafted after longer periods of expansion (6 and 11 days), and at different cell concentrations (2.5×10^5 to 1×10^6, healthy control 1 line) (n = 6 mice) (data not shown). Quantification of the number of $Spectrin^+$ fibers showed that cell engraftment efficiency was 35–58 fibers/section, with 87–127 Lamin A/C^+ nuclei/section (Figure 6B, using two independent cell lines: control 1 and control 5). Lamin A/C^+ nuclei were found within myofibers and in the interstitium. A subset of Lamin A/C^+ nuclei was found at a satellite cell position (Figure S3B top); however, very few of those were $Pax7^+$ (Figure S3B bottom). The location of Lamin A/C^+ nuclei was as follows: ~45% was found within human $Spectrin^+$ myofibers, suggesting that these contributed to myofiber formation (Figure 6C); 25%–36% was found in the interstitium (Figure S3C); the remaining 23%–40% was found within $Spectrin^-$ myofibers, which may indicate that in those (multinucleated) fibers mouse nuclei were dominant. These results demonstrate the engraftment potential and regenerative capacities of expanded myogenic progenitors and their participation in muscle regeneration *in vivo*.

DISCUSSION

In this study, we have characterized FACS-purified myogenic progenitors for their applicability *in vitro* and *in vivo*, and provide a detailed protocol to generate these cells. The principle of the procedure and its possible applications are shown in Figure 7. We showed that it is possible to reproducibly generate myogenic progenitors from 15

Figure 4. Molecular Profiling and Purity of Myogenic Progenitors

(A) Purified myogenic progenitors have a myogenic gene expression signature. Heatmap showing a comparison of genome-wide mRNA expression (as measured by RNA-seq) from myogenic progenitors and publicly available datasets. Purified myogenic progenitors from two healthy control iPSCs were included: cells were either expanded for ~15 days in proliferation medium or differentiated for 4 days. Published datasets are listed in Table S3. Datasets were analyzed using the "new Tuxedo" pipeline as described in Pertea et al. (2016). Spearman correlations are shown. Datasets generated in this study are indicated in green.

(B) Purified myogenic progenitors do not express pluripotency markers (*POU5F1*, *LIN28A*, and *NANOG*), neuronal markers (*SOX2*, *GFAP*, and *MAP2*), or chondrogenic markers (*SOX5*, *COMP*, and *ACAN*). Data were extracted from (A). Data are means ± SD of two independent cultures per cell line.

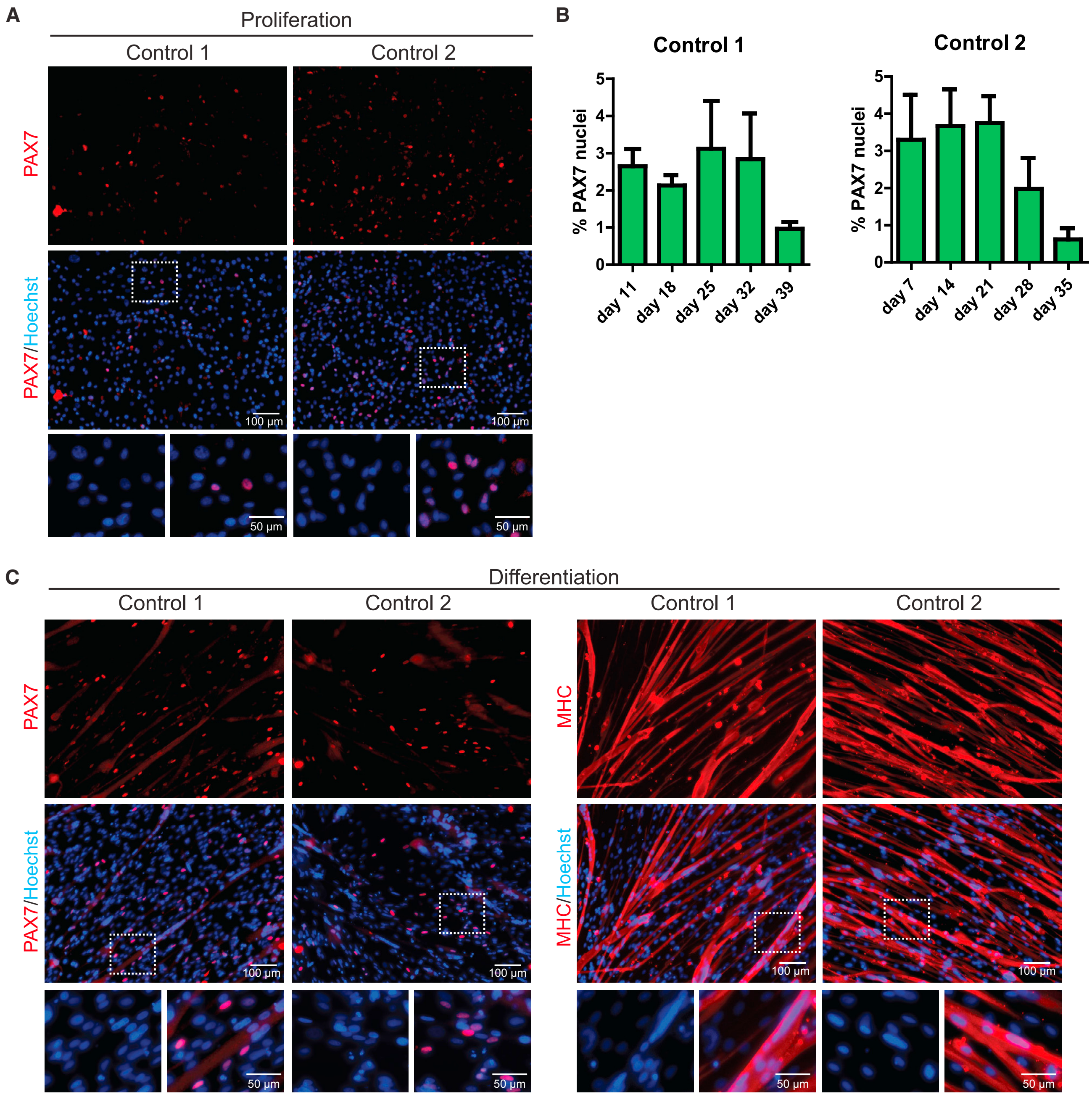

Figure 5. PAX7 Expression during *In Vitro* Proliferation and Differentiation of Purified Myogenic Progenitors

(A) Purified myogenic progenitors from two healthy control iPSCs were expanded for ~25 days in proliferation medium and stained with a PAX7 antibody and Hoechst to stain nuclei.

(B) Quantification of $PAX7^{+}$ cells during expansion of myogenic progenitors from the two healthy control iPSCs shown in (A). Data are means ± SD of n = 5 fields per point.

(C) Myogenic progenitors were differentiated for 6 days to myotubes. Immunofluorescent analysis was performed using a PAX7 antibody (in red) or an MHC antibody (in red) to monitor myotube formation, as indicated. Nuclei were stained with Hoechst (blue).

iPSC lines that were derived from different donors. As we have shown previously, 4×10^4 sorted myogenic progenitors could be expanded to as much as 1×10^{12} cells within 31 days without losing differentiation capacity (van der Wal et al., 2017b). Our current data show that the period during which myogenic progenitors can be expanded can

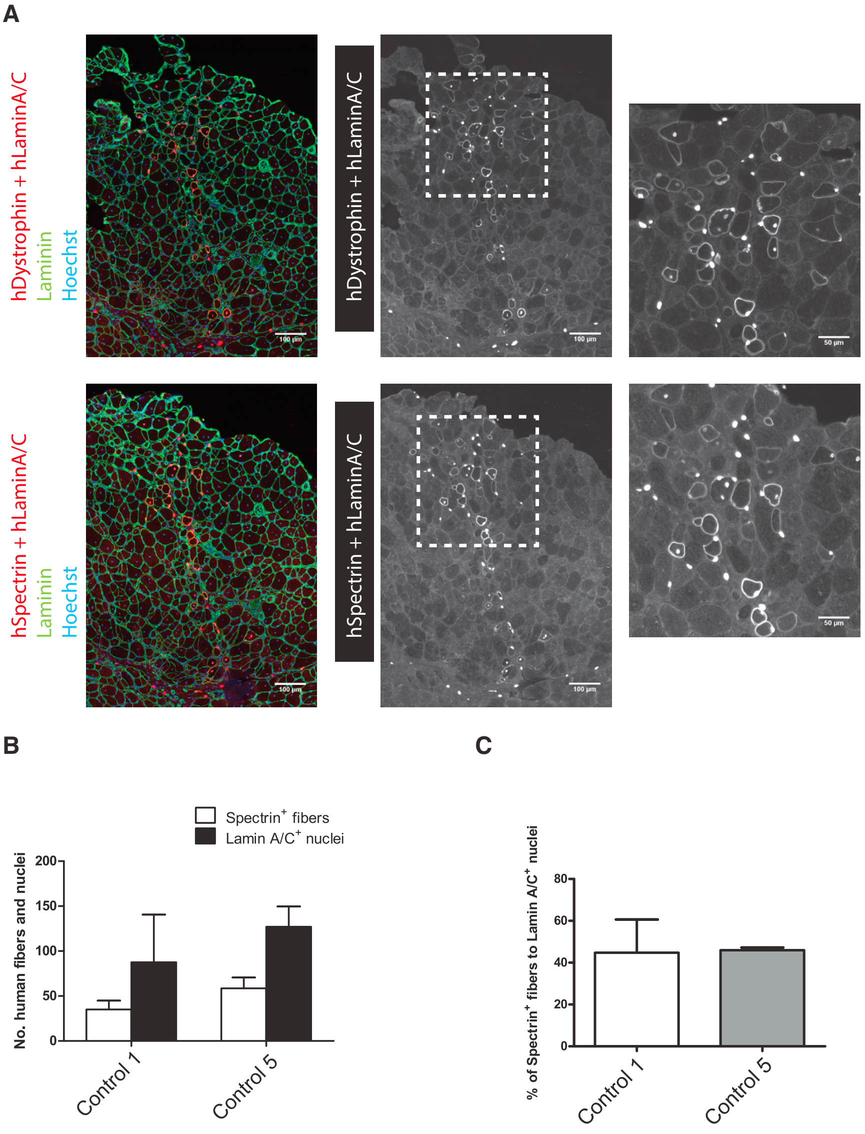

Figure 6. *In Vivo* Myogenic Potential of Purified Myogenic Progenitors Following Engraftment in Immunodeficient Mice

(A) Twenty-four hours before transplantation, the TA of NSG mice was injured using $BaCl_2$. Myogenic progenitors were administered using intramuscular injection of 5×10^5 cells. Four weeks after transplantation, engraftment was determined by immunohistochemistry of human-specific Lamin A/C and Dystrophin or Spectrin (white or red) and multi-species Laminin (green) on consecutive cross sections.

(B and C) (B) Quantification of Spectrin$^+$ muscle fibers and Lamin A/C$^+$ nuclei and (C) the percentage of Spectrin$^+$ fibers relative to the total number of Lamin A/C$^+$ nuclei per section of each biological replicate. Data in (B) and (C) are means ± SD (n = 2 TAs transplanted per line used. Each replicate was transplanted in different mice). All sections were counterstained with Hoechst (blue). Scale bars represent 100 μm, and 50 μm on insets.

be extended to up to 43 days. After ~50 days of expansion, changes in morphology and proliferation rate suggested the initiation of a senescent phenotype. It is therefore likely that, during the expansion, myogenic progenitors slowly progress to a myoblast-like phenotype, a cell type that is known to undergo replicative senescence during passaging (Bigot et al., 2008). After 43 days of culture, myogenic progenitors had expanded as much as 5×10^{11}-fold (the maximum value obtained), allowing the generation of at least 2×10^{16} cells, which should be sufficient for subsequent analyses, including high-throughput screenings and engraftment studies.

We generated a generic donor construct that can be used for precise and highly efficient gene correction. The selection of positive clones is facilitated by its inclusion of a selection marker. The option of removing the selection marker using transient CRE recombinase expression may be useful in future *in vitro* and *in vivo* applications. A prerequisite for the strategy of inserting a wild-type copy of a cDNA of interest is that overexpression of the transgene product should not be harmful; if it is, the choice of promoter that drives the transgene should be optimized. Overexpression of a transgene is expected to benefit the development of cell-based therapeutic strategies. In the case of skeletal muscle, which consists of multinucleated cells, it can be envisioned that overexpression in a subset of engrafted myonuclei that become part of the syncytium of the affected myofibers would cross-correct part of the myofiber. If the transgene product is secreted, as GAA is secreted in Pompe disease, overexpression potentially

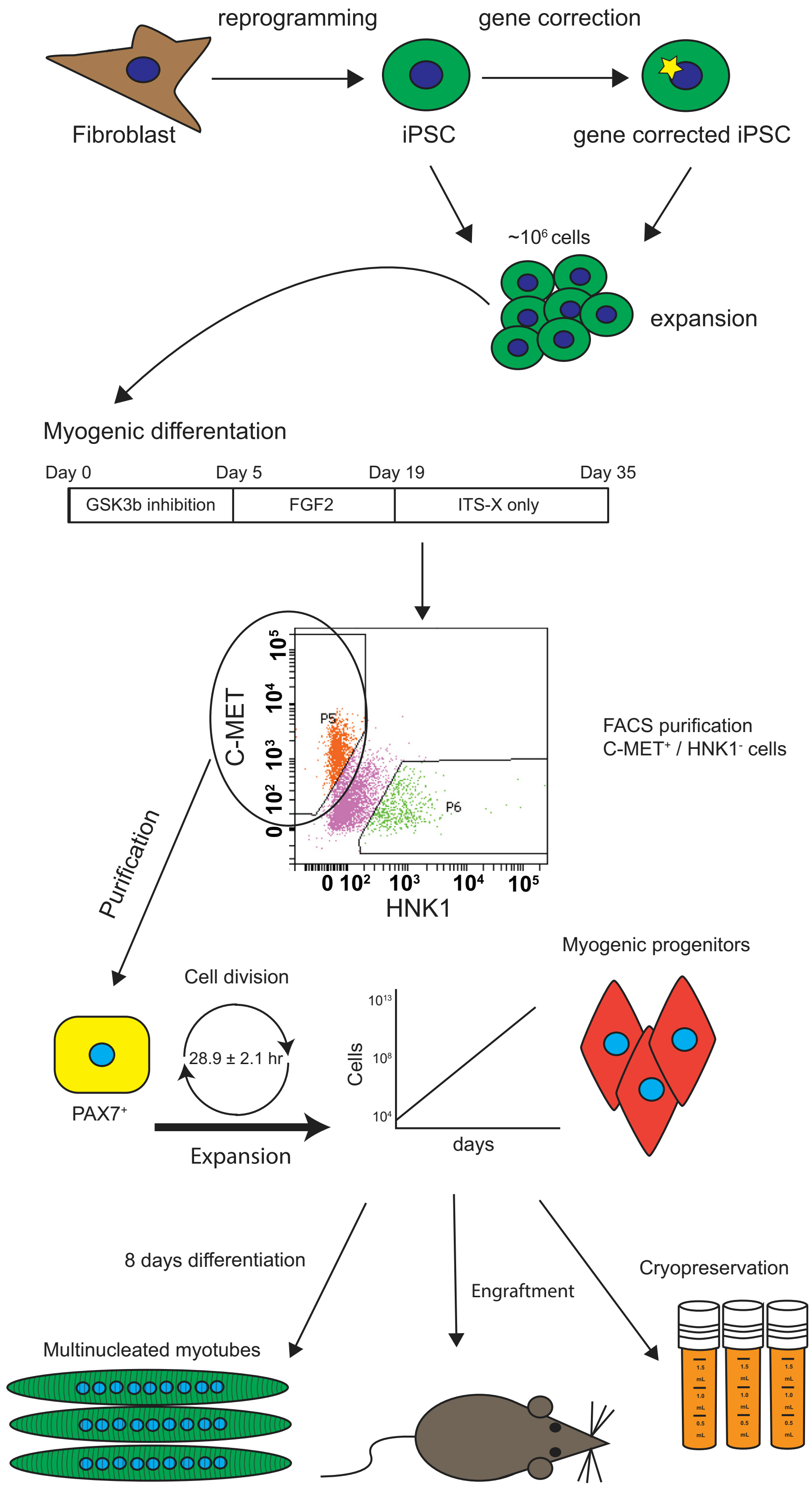

Figure 7. Cartoon Highlighting the Applications of Myogenic Progenitors Described Here

Human iPSCs derived from healthy controls or patients are used as starting cells. Gene correction is applied to iPSCs using CRISPR/Cas9-mediated insertion of a cDNA into a safe harbor. Original or gene-corrected iPSCs are differentiated into the myogenic lineage using a 35-day transgene-free protocol. Myogenic progenitors are purified using a 1-step FACS procedure, and are then expanded (up to 5×10^{11}-fold) and cryopreserved. During expansion, purified myogenic progenitors are differentiated *in vitro* into myotubes with high fusion index, and show striation and spontaneous contraction upon *in vitro* maturation. Upon engraftment in immunodeficient mice, purified and expanded myogenic progenitors form human mononuclear cells and contribute to myofiber formation *in vivo*.

results in cross-correction of neighboring myofibers (Zaretsky et al., 1997).

Both iPSC-derived myogenic progenitors and SCs express PAX7 during *in vitro* proliferation and differentiation, and contribute to myofiber formation after intramuscular engraftment in immunodeficient mice *in vivo*. We showed that, upon optimization of the differentiation process using defined medium conditions, the cells not only expressed fast MHC, α-actinin, and titin but also formed functional sarcomeres, thereby allowing spontaneous contractions. These data revealed enhanced maturation compared with that found in our previous report (van der Wal et al., 2017b) and showed that it is possible to generate mature myofibers from purified iPSC-derived cultures. The formation of myogenic progenitors from Pompe patient-derived iPSCs was not hampered by the underlying disorder, and we expect that this approach can be used to model other disorders that affect muscle cells. It remains to be determined whether, as Chal et al. (2015) report, purified myogenic progenitors have the capacity to differentiate into millimeter-long skeletal muscle cells with $PAX7^+$ cells embedded between the sarcolemma and the basal lamina.

It is essential for the development of stem cell-based therapies that the transplantable cell preparations are highly pure and well characterized before transplantation in human patients can be considered. The development of techniques for the expansion and manipulation of pure myogenic progenitor populations *ex vivo* is therefore critical to the further development of this field. In this paper we have provided evidence for the successful engraftment of myogenic progenitors in pre-injured muscles of mice over a period of 4 weeks post-transplantation. The efficiencies of engraftment of mononuclear cells and their contribution to myofibers were comparable with those recently obtained using inducible PAX7 overexpression (Magli et al., 2017). Transplanted myogenic progenitors demonstrated their ability to regenerate injured muscle, as was shown by the detection of centrally located lamin A/C^+ human nuclei, a characteristic perceived only in fusion-competent myoblasts.

Future studies should identify the stem cell properties of transplanted human myogenic progenitors that allow transplanted donor cells to make a long-term contribution to muscle regeneration. This would provide researchers with novel tools that would help them make progress in the development of muscle stem cell therapies for treating muscle-wasting diseases.

EXPERIMENTAL PROCEDURES

Ethics Approval and Consent to Participate

The Institutional Review Board approved the study protocol, and all patients provided written informed consent. All animal experiments were approved by the animal experiments committee DEC-Consult.

Culture of Myogenic Progenitors

Myogenic progenitors were expanded in myogenitor progenitor proliferation medium consisting of DMEM high glucose (Gibco, Waltham, MA) supplemented with 10% fetal bovine serum (Hyclone, Thermo Scientific, Waltham, MA), 1% penicillin-streptomycin-glutamine (P/S/G) (Gibco, Waltham, MA), and 100 ng/mL FGF2 (Prepotech, Rocky Hill, NJ) on extracellular matrix-coated dishes (1:200 diluted, Sigma-Aldrich, E6909). For splitting, myogenic progenitors were detached with TrypLe reagent (Gibco, Waltham, MA) diluted 2× with PBS (Gibco, Waltham, MA). For cryopreservation, myogenic progenitors were detached as described above, and after centrifugation the cell pellet was resuspended in myogenic progenitor proliferation medium supplemented with 10% DMSO. Standard cell culture techniques were used for the freeze and thaw procedure.

RNA Isolation and RNA-Seq

Myogenic progenitors were expanded for ~15 days and harvested either in proliferation conditions or after 4 days of differentiation as described previously (van der Wal et al., 2017b). RNA was extracted using the RNeasy minikit with DNase treatment (QIAGEN, Germantown, MD). Sequencing libraries were prepared using TruSeq Stranded mRNA Library Prep Kit (Illumina, San Diego, CA) according to the manufacturer's instructions. Libraries were sequenced on a HiSeq2500 sequencer (Illumina, San Diego, CA) in rapid-run mode according to the manufacturer's instructions. Reads 50 bp in length were generated. The RNA-seq datasets listed in Table S3 were downloaded and aligned with the datasets generated in this study using the new Tuxedo pipeline as described by Pertea et al. (2016). Shortly, RNA-seq data were aligned using Hisat2 (version 2.1.0) to hg38 from University of California, Santa Cruz. The alignments were converted to BAM format using Samtools (version 1.3.1). Then, StringTie was used to quantify transcript expression levels according to the reference transcripts. For KEGG analysis, gene expression was quantified using StringTie with the -e option.

Maturation of Myogenic Progenitors into Skeletal Muscle Cells

When myogenic progenitors reached 90% confluence, cells were switched to myogenic progenitor differentiation medium containing DMEM high glucose supplemented with 1% P/S/G, 1× ITS-X, and 1% knockout serum replacement (all Gibco). Medium was not refreshed during differentiation and cells were harvested at 6 days, 8 days, or 12 days.

Construction of Donor Vector

To generate the generic donor vector for the overexpression of the gene of interest via CRISPR/Cas9-mediated knockin, we used the pCAGEN and pEF-GFP vectors (available on addgene: #11160 and #11154) as starting points. The neomycin selection cassette was introduced via PCR amplification into the pCAGEN vector, destroying the EcoRI and NotI sites. KpnI and ClaI sites were then added to the SalI site, and loxP and SfuI sites were added to the HindIII site. In the pEF-GFP vector, a KpnI site was added to the SalI site and loxP and ClaI sites were added to the HindIII site. Vectors were

combined using the KpnI and ClaI sites. The *GAA* cDNA was introduced via PCR amplification with EcoRI and NotI fragments. The 5′ homology arm of 700 bp was added via PCR amplification with KpnI fragments, and the 3′ homology arm of 972 bp was added via PCR amplification with HindIII fragments. All constructs were validated by sequencing. Cloning details are available on request.

Glycogen Assay

Myogenic progenitors were differentiated for 6 days in myogenic progenitor differentiation medium. On day 5 of differentiation, skeletal muscle cells were starved with differentiation medium without glucose (DMEM no glucose, Gibco). On day 6, skeletal muscle cells were detached with a scraper and the pellet was lysed with ice-cold protein lysis buffer (see Supplemental Experimental Procedures). Glycogen was measured as described in Bergsma et al. (2015).

Gene Editing of iPSCs

To select optimal target sites for the AAVS1 locus, single guide RNA (sgRNA) sequences were designed using the CRISPRscan program (Moreno-Mateos et al., 2015). The sgRNA CCACTAGGGACAGGATTGGTGA was expressed from a TOPO vector containing the U6 promoter (addgene: 41824). Confluent iPSCs on feeders were pretreated 4 hr before nucleofection with 10 μM Rock inhibitor (Y-27632 dihydrochloride, Ascent Scientific, Asc-129). Single cells were generated from iPSC colonies by incubating with Accutase (Thermo Scientific, Waltham, MA), and 2×10^6 cells were nucleofected with 4 μg of pCAG-hCAS9-GFP (addgene: 44719), 3 μg of TOPO-sgRNA, and 2 μg of donor vector using Amaxa Human Stem Cell Nucleofector Kit2 (VPH-5022, Lonza, Walkersville, MD) with program B-016. After nucleofection, cells were recovered in iPSC-conditioned medium (iPSC medium incubated for 24 hr on feeder cells) supplemented with 20 ng/mL FGF2 (Prepotech, Rocky Hill, NJ) and 10 μM rock inhibitor. iPSCs were selected after 48 hr of nucleofection with 100 μg/mL G-418 (Invivogen, San Diego, CA). Approximately 14 days after selection of the iPSCs, single colonies were picked and genotyped using primers from Table S4.

Transplantation into NSG Mice

NSG (Jackson Laboratories) mice aged 2–6 months were used for transplantation studies. Mice (independently of gender) were anesthetized with isoflurane in oxygen from a vaporizer. Regeneration of skeletal muscle was induced by chemical injury. The endogenous skeletal muscle fibers of the mice were injured by injection with 50 μL of 1.2% barium chloride ($BaCl_2$) into the TA muscle. Twenty-four hours later, 20 μL of 5×10^5 dissociated cells were injected into the TA muscle in duplicates (one female and one male). Transplanted cells in this study were expanded for 3 days. PBS-injected TAs were used as negative control for cell transplantations. Mice were sacrificed 4 weeks after cell transplantation, and their TA muscles harvested. TA muscles were frozen in isopentane cooled in liquid nitrogen and stored at −80°C until analysis; 10 μm cryosections were obtained at intervals throughout the entire muscle and were either stored at −80°C for further immunostaining or were used immediately for PAX7 staining.

Immunofluorescent Stainings

Muscle cryosections were fixed in ice-cold acetone for 5 min, followed by a permeabilization step with 0.3% Triton X-100 in PBS for 20 min. Samples were incubated with a blocking solution of 20% goat serum (DAKO, Santa Clara, CA) and 2% BSA (Sigma-Aldrich, Irvine, UK) in 0.1% Tween in PBS for 1 hr. Sections were incubated with primary antibodies mouse anti-human Lamin A/C (1:100, VP-L550, Vector Laboratories, Burlingame, CA) plus mouse anti-human Spectrin (1:100, SPEC1-CE, Leica, Wetzlar, Germany) or mouse anti-human Dystrophin (1:150, MABT827, Millipore) co-stained with rabbit anti-Laminin (1:100, L9393, Sigma-Aldrich, Irvine, UK) overnight at 4°C. Tissue sections were stained with secondary antibodies goat anti-rabbit (Alexa Fluor 488, 1:500, A-21141, Life Technologies, Carlsbad, CA) and horse anti-mouse biotin (1:250, BA-2000, Vector Laboratories, Burlingame, CA) for 1 hr at room temperature, followed by incubation with Streptavidin 594 (1:500, S-32356, Invitrogen, Carlsbad, CA) for 30 min. Freshly cut tissue was used for PAX7 stainings. Sections were fixed in 4% paraformaldehyde for 5 min and blocked with 20% goat serum and 2% BSA in 0.5% Triton X-100 in PBS for 1 hr, then incubated with mouse anti-PAX7 (1/20, DSHB), Lamin A/C, and Laminin in blocking solution for 2 hr at room temperature. Goat anti-mouse IgG1 Cy3 (1:500, 115-165-205, Jackson ImmunoResearch), goat anti-mouse IgG2b Alexa Fluor 488 (1:500, A-21141, Thermo Fisher), and goat anti-rabbit Alexa Fluor 647 (1:500, A21245, Invitrogen, Carlsbad, CA) were used in 0.1% PBST for 1 hr. All sections were incubated with Hoechst nuclear staining (1:15,000 Invitrogen, Carlsbad, CA) for 10 min and mounted with Mowiol medium (Sigma-Aldrich, Irvine, UK). Images were obtained using confocal microscopy (Zeiss LSM 700).

Statistical Analysis

Data represent mean ± SD, and p values refer to two-sided t tests. Multiple groups were tested with one-way ANOVA followed by individual two-sided t tests. A p value of <0.05 was considered to be significant. Data showed normal variance and no samples were excluded from the analysis. Images for quantification were randomly selected.

ACCESSION NUMBERS

RNA-seq fastq files and data are accessible at GEO under GEO: GSE111163.

SUPPLEMENTAL INFORMATION

Supplemental Information includes Supplemental Experimental Procedures, three figures, five tables, and two videos and can be found with this article online at https://doi.org/10.1016/j.stemcr.2018.04.002.

AUTHOR CONTRIBUTIONS

Myogenic protocol, E.v.d.W., S.i.G., T.J.M.v.G., and W.W.M.P.P.; Engraftment, P.H.-H., T.J.M.v.G., G.J.S., and W.W.M.P.P.; Expression analysis, R.W., T.H.C., W.F.J.v.I., E.v.d.W., and W.W.M.P.P.; Gene edit and pathology, M.B., E.v.d.W., S.i.G., and W.W.M.P.P.; Funding, W.W.M.P.P., A.T.v.d.P., and G.J.S.; Data interpretation,

all authors; Writing, E.v.d.W., P.H.-H., and W.W.M.P.P.; Supervision, G.J.S., T.H.C., and W.W.M.P.P.

ACKNOWLEDGMENTS

We thank Dr. Tiziano Barberi for discussion; Dr. Schambach for the OSKM-lentiviral vector; and Dr. Christian Freund, Prof. Dr. Christine Mummery, Dr. Mehrnaz Ghazvini, and Prof. Dr. Joost Gribnau for providing healthy control fibroblasts and healthy control iPSC lines. This work was funded by the Prinses Beatrix Spierfonds/Stichting Spieren voor Spieren (grant W.OR13-21), Tex Net, and the Croucher Innovation Award (to T.H.C.). A.T.v.d.P. has provided consulting services for various industries in the field of Pompe disease under an agreement between these industries and Erasmus MC, Rotterdam, the Netherlands. All the other authors declare no conflict of interest.

Received: September 24, 2017
Revised: March 31, 2018
Accepted: April 3, 2018
Published: May 3, 2018

REFERENCES

Baghdadi, M.B., and Tajbakhsh, S. (2017). Regulation and phylogeny of skeletal muscle regeneration. Dev. Biol. *433*, 200–209.

Bergsma, A.J., Kroos, M., Hoogeveen-Westerveld, M., Halley, D., van der Ploeg, A.T., and Pijnappel, W.W. (2015). Identification and characterization of aberrant GAA pre-mRNA splicing in Pompe disease using a generic approach. Hum. Mutat. *36*, 57–68.

Bigot, A., Jacquemin, V., Debacq-Chainiaux, F., Butler-Browne, G.S., Toussaint, O., Furling, D., and Mouly, V. (2008). Replicative aging down-regulates the myogenic regulatory factors in human myoblasts. Biol. Cell *100*, 189–199.

Borchin, B., Chen, J., and Barberi, T. (2013). Derivation and FACS-mediated purification of PAX3+/PAX7+ skeletal muscle precursors from human pluripotent stem cells. Stem Cell Reports *1*, 620–631.

Bursac, N., Juhas, M., and Rando, T.A. (2015). Synergizing engineering and biology to treat and model skeletal muscle injury and disease. Annu. Rev. Biomed. Eng. *17*, 217–242.

Caron, L., Kher, D., Lee, K.L., McKernan, R., Dumevska, B., Hidalgo, A., Li, J., Yang, H., Main, H., Ferri, G., et al. (2016). A human pluripotent stem cell model of facioscapulohumeral muscular dystrophy-affected skeletal muscles. Stem Cells Transl. Med. *5*, 1145–1161.

Chal, J., Al Tanoury, Z., Hestin, M., Gobert, B., Aivio, S., Hick, A., Cherrier, T., Nesmith, A.P., Parker, K.K., and Pourquie, O. (2016). Generation of human muscle fibers and satellite-like cells from human pluripotent stem cells in vitro. Nat. Protoc. *11*, 1833–1850.

Chal, J., Oginuma, M., Al Tanoury, Z., Gobert, B., Sumara, O., Hick, A., Bousson, F., Zidouni, Y., Mursch, C., Moncuquet, P., et al. (2015). Differentiation of pluripotent stem cells to muscle fiber to model Duchenne muscular dystrophy. Nat. Biotechnol. *33*, 962–969.

Charville, G.W., Cheung, T.H., Yoo, B., Santos, P.J., Lee, G.K., Shrager, J.B., and Rando, T.A. (2015). Ex vivo expansion and in vivo self-renewal of human muscle stem cells. Stem Cell Reports *5*, 621–632.

Choi, I.Y., Lim, H., Estrellas, K., Mula, J., Cohen, T.V., Zhang, Y., Donnelly, C.J., Richard, J.P., Kim, Y.J., Kim, H., et al. (2016). Concordant but varied phenotypes among Duchenne muscular dystrophy patient-specific myoblasts derived using a human iPSC-based model. Cell Rep. *15*, 2301–2312.

Darabi, R., Arpke, R.W., Irion, S., Dimos, J.T., Grskovic, M., Kyba, M., and Perlingeiro, R.C. (2012). Human ES- and iPS-derived myogenic progenitors restore DYSTROPHIN and improve contractility upon transplantation in dystrophic mice. Cell Stem Cell *10*, 610–619.

Dumont, N.A., Bentzinger, C.F., Sincennes, M.C., and Rudnicki, M.A. (2015). Satellite cells and skeletal muscle regeneration. Compr. Physiol. *5*, 1027–1059.

Golding, J.P., Calderbank, E., Partridge, T.A., and Beauchamp, J.R. (2007). Skeletal muscle stem cells express anti-apoptotic ErbB receptors during activation from quiescence. Exp. Cell Res. *313*, 341–356.

Hockemeyer, D., and Jaenisch, R. (2016). Induced pluripotent stem cells meet genome editing. Cell Stem Cell *18*, 573–586.

Kaplan, J.C., and Hamroun, D. (2015). The 2016 version of the gene table of monogenic neuromuscular disorders (nuclear genome). Neuromuscul. Disord. *25*, 991–1020.

Kim, J., Magli, A., Chan, S.S.K., Oliveira, V.K.P., Wu, J., Darabi, R., Kyba, M., and Perlingeiro, R.C.R. (2017). Expansion and purification are critical for the therapeutic application of pluripotent stem cell-derived myogenic progenitors. Stem Cell Reports *9*, 12–22.

Lepper, C., Partridge, T.A., and Fan, C.M. (2011). An absolute requirement for Pax7-positive satellite cells in acute injury-induced skeletal muscle regeneration. Development *138*, 3639–3646.

Lombardo, A., Cesana, D., Genovese, P., Di Stefano, B., Provasi, E., Colombo, D.F., Neri, M., Magnani, Z., Cantore, A., Lo Riso, P., et al. (2011). Site-specific integration and tailoring of cassette design for sustainable gene transfer. Nat. Methods *8*, 861–869.

Magli, A., Incitti, T., Kiley, J., Swanson, S.A., Darabi, R., Rinaldi, F., Selvaraj, S., Yamamoto, A., Tolar, J., Yuan, C., et al. (2017). PAX7 targets, CD54, integrin alpha9beta1, and SDC2, allow isolation of human ESC/iPSC-derived myogenic progenitors. Cell Rep. *19*, 2867–2877.

Mauro, A. (1961). Satellite cell of skeletal muscle fibers. J. Biophys. Biochem. Cytol. *9*, 493–495.

Moreno-Mateos, M.A., Vejnar, C.E., Beaudoin, J.D., Fernandez, J.P., Mis, E.K., Khokha, M.K., and Giraldez, A.J. (2015). CRISPRscan: designing highly efficient sgRNAs for CRISPR-Cas9 targeting in vivo. Nat. Methods *12*, 982–988.

Murphy, M.M., Lawson, J.A., Mathew, S.J., Hutcheson, D.A., and Kardon, G. (2011). Satellite cells, connective tissue fibroblasts and their interactions are crucial for muscle regeneration. Development *138*, 3625–3637.

Pertea, M., Kim, D., Pertea, G.M., Leek, J.T., and Salzberg, S.L. (2016). Transcript-level expression analysis of RNA-seq experiments with HISAT, StringTie and Ballgown. Nat. Protoc. *11*, 1650–1667.

Sambasivan, R., Yao, R., Kissenpfennig, A., Van Wittenberghe, L., Paldi, A., Gayraud-Morel, B., Guenou, H., Malissen, B., Tajbakhsh, S., and Galy, A. (2011). Pax7-expressing satellite cells are indispensable for adult skeletal muscle regeneration. Development *138*, 3647–3656.

Shelton, M., Kocharyan, A., Liu, J., Skerjanc, I.S., and Stanford, W.L. (2016). Robust generation and expansion of skeletal muscle progenitors and myocytes from human pluripotent stem cells. Methods *101*, 73–84.

Shelton, M., Metz, J., Liu, J., Carpenedo, R.L., Demers, S.P., Stanford, W.L., and Skerjanc, I.S. (2014). Derivation and expansion of PAX7-positive muscle progenitors from human and mouse embryonic stem cells. Stem Cell Reports *3*, 516–529.

Soldner, F., Laganiere, J., Cheng, A.W., Hockemeyer, D., Gao, Q., Alagappan, R., Khurana, V., Golbe, L.I., Myers, R.H., Lindquist, S., et al. (2011). Generation of isogenic pluripotent stem cells differing exclusively at two early onset Parkinson point mutations. Cell *146*, 318–331.

Swartz, E.W., Baek, J., Pribadi, M., Wojta, K.J., Almeida, S., Karydas, A., Gao, F.B., Miller, B.L., and Coppola, G. (2016). A novel protocol for directed differentiation of C9orf72-associated human induced pluripotent stem cells into contractile skeletal myotubes. Stem Cells Transl. Med. *5*, 1461–1472.

Takahashi, K., and Yamanaka, S. (2016). A decade of transcription factor-mediated reprogramming to pluripotency. Nat. Rev. Mol. Cell Biol. *17*, 183–193.

Theret, M., Gsaier, L., Schaffer, B., Juban, G., Ben Larbi, S., Weiss-Gayet, M., Bultot, L., Collodet, C., Foretz, M., Desplanches, D., et al. (2017). AMPKalpha1-LDH pathway regulates muscle stem cell self-renewal by controlling metabolic homeostasis. EMBO J. *36*, 1946–1962.

van der Ploeg, A.T., and Reuser, A.J. (2008). Pompe's disease. Lancet *372*, 1342–1353.

van der Wal, E., Bergsma, A.J., Pijnenburg, J.M., van der Ploeg, A.T., and Pijnappel, W. (2017a). Antisense oligonucleotides promote exon inclusion and correct the common c.-32-13T>G GAA splicing variant in Pompe disease. Mol. Ther. Nucleic Acids *7*, 90–100.

van der Wal, E., Bergsma, A.J., van Gestel, T.J.M., In 't Groen, S.L.M., Zaehres, H., Arauzo-Bravo, M.J., Scholer, H.R., van der Ploeg, A.T., and Pijnappel, W. (2017b). GAA deficiency in Pompe disease is alleviated by exon inclusion in iPSC-derived skeletal muscle cells. Mol. Ther. Nucleic Acids *7*, 101–115.

Xu, C., Tabebordbar, M., Iovino, S., Ciarlo, C., Liu, J., Castiglioni, A., Price, E., Liu, M., Barton, E.R., Kahn, C.R., et al. (2013). A zebrafish embryo culture system defines factors that promote vertebrate myogenesis across species. Cell *155*, 909–921.

Zaretsky, J.Z., Candotti, F., Boerkoel, C., Adams, E.M., Yewdell, J.W., Blaese, R.M., and Plotz, P.H. (1997). Retroviral transfer of acid alpha-glucosidase cDNA to enzyme-deficient myoblasts results in phenotypic spread of the genotypic correction by both secretion and fusion. Hum. Gene Ther. *8*, 1555–1563.

Stem Cell Reports, Volume *10*

Supplemental Information

Large-Scale Expansion of Human iPSC-Derived Skeletal Muscle Cells for Disease Modeling and Cell-Based Therapeutic Strategies

Erik van der Wal, Pablo Herrero-Hernandez, Raymond Wan, Mike Broeders, Stijn L.M. in 't Groen, Tom J.M. van Gestel, Wilfred F.J. van IJcken, Tom H. Cheung, Ans T. van der Ploeg, Gerben J. Schaaf, and W.W.M. Pim Pijnappel

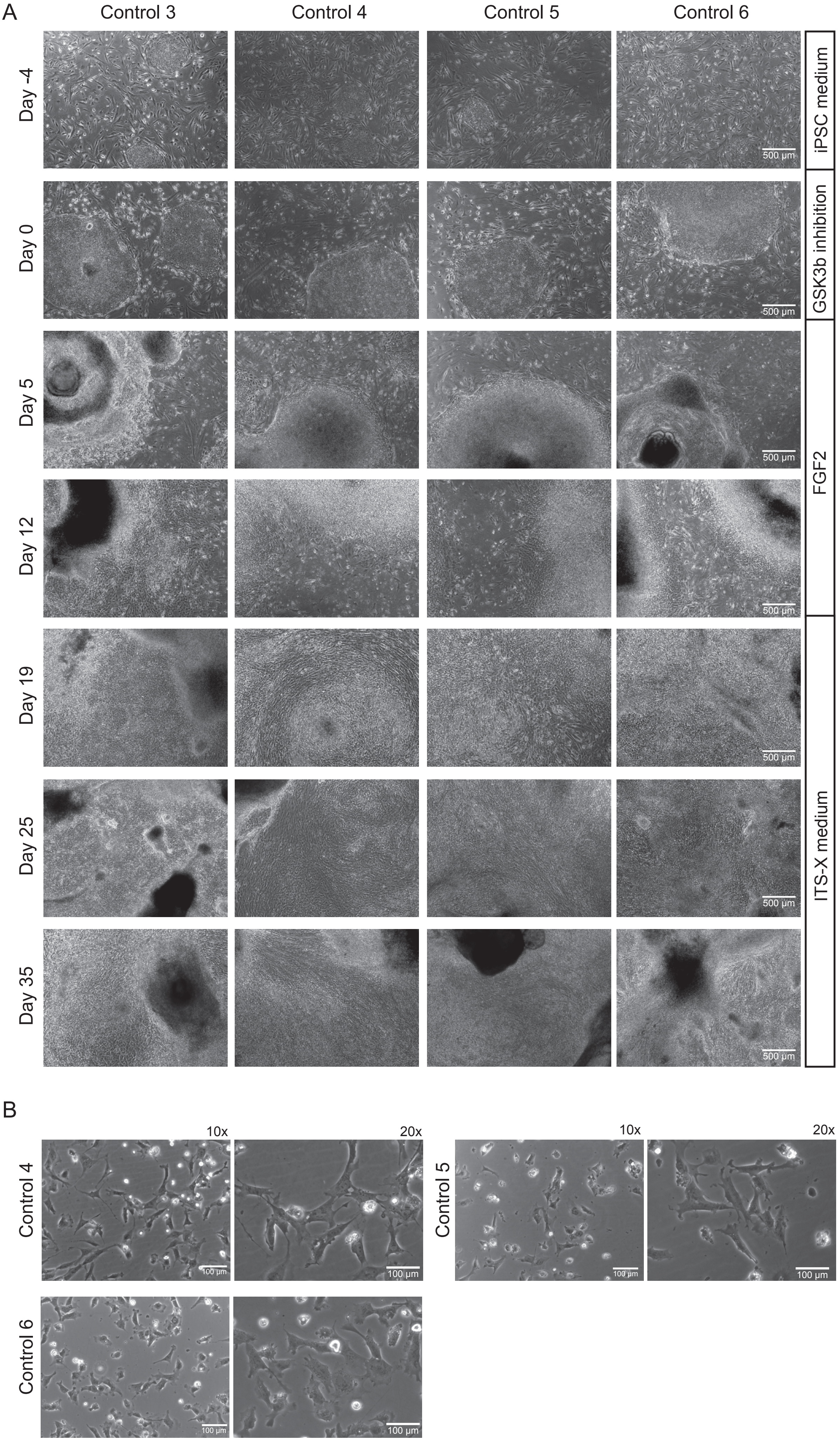
A
Control 3
Control 4
Control 5
Control 6
Day -4
Day 0
Day 5
Day 12
Day 19
Day 25
Day 35
iPSC medium
GSK3b inhibition
FGF2
ITS-X medium
500 µm
B
10x
20x
Control 4
Control 5
Control 6
100 µm

Figure S1

A

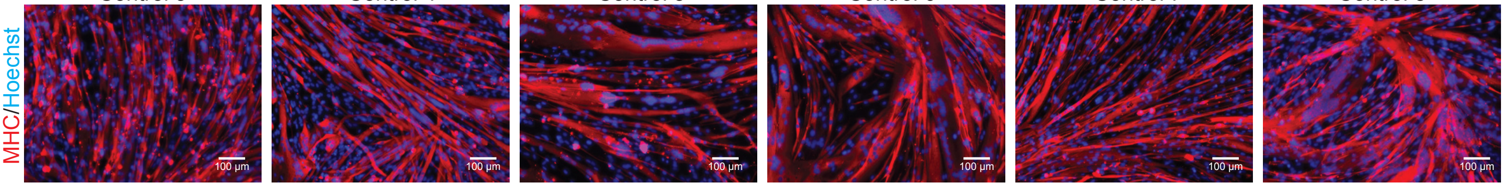

	Control 3 average	Control 3 sd	Control 4 average	Control 4 sd	Control 5 average	Control 5 sd	Control 6 average	Control 6 sd	Control 7 average	Control 7 sd	Control 8 average	Control 8 sd
**5-14d**	59,1%	5,3%	51,4%	3,9%	52,9%	7,0%	39,2%	11,8%	63,1%	1,7%	91,6%	4,4%
**12-18d**	69,4%	4,8%	37,3%	1,4%	37,9%	12,1%	40,1%	11,1%	51,8%	14,4%	62,7%	13,4%
**20-25d**	54,3%	16,2%	46,0%	8,6%	57,9%	7,8%	81,9%	6,3%	48,4%	20,5%	50,8%	2,3%
**27-35d**	31,4%	6,8%	49,5%	7,0%	64,1%	3,6%	79,6%	4,0%	35,3%	1,7%	41,7%	4,3%

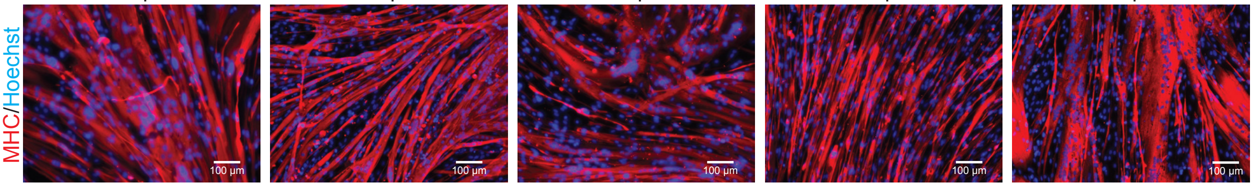

	Pompe 3 average	Pompe 3 sd	Pompe 4 average	Pompe 4 sd	Pompe 5 average	Pompe 5 sd	Pompe 6 average	Pompe 6 sd	Pompe 7 average	Pompe 7 sd
**5-14d**	53,6%	27,0%	54,1%	8,2%	44,8%	10,4%	43,3%	7,8%	33,0%	7,7%
**12-18d**	45,8%	9,7%	42,3%	4,5%	44,3%	7,3%	49,4%	4,1%	52,5%	1,3%
**20-25d**	77,2%	13,7%	49,8%	7,0%	59,0%	19,2%	36,8%	8,3%	53,4%	6,6%
**27-35d**	65,1%	12,9%	37,9%	8,1%	43,6%	2,1%	35,5%	8,7%	38,7%	6,7%

B Control 2

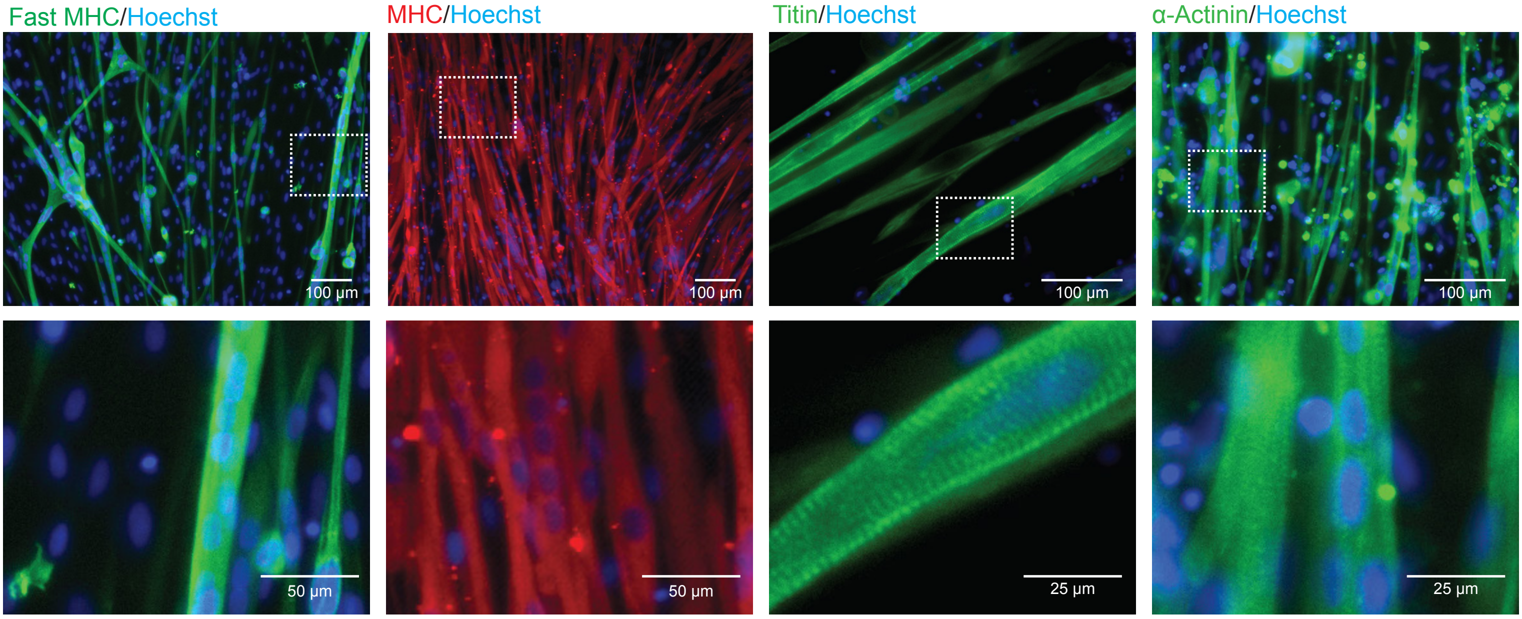

C

KEGG pathway analysis: MPCs vs Activated muscle stem cells

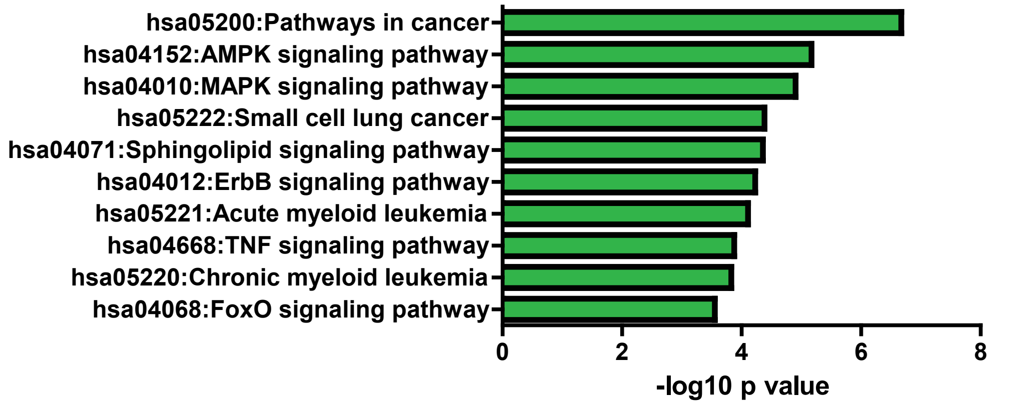

Figure S2

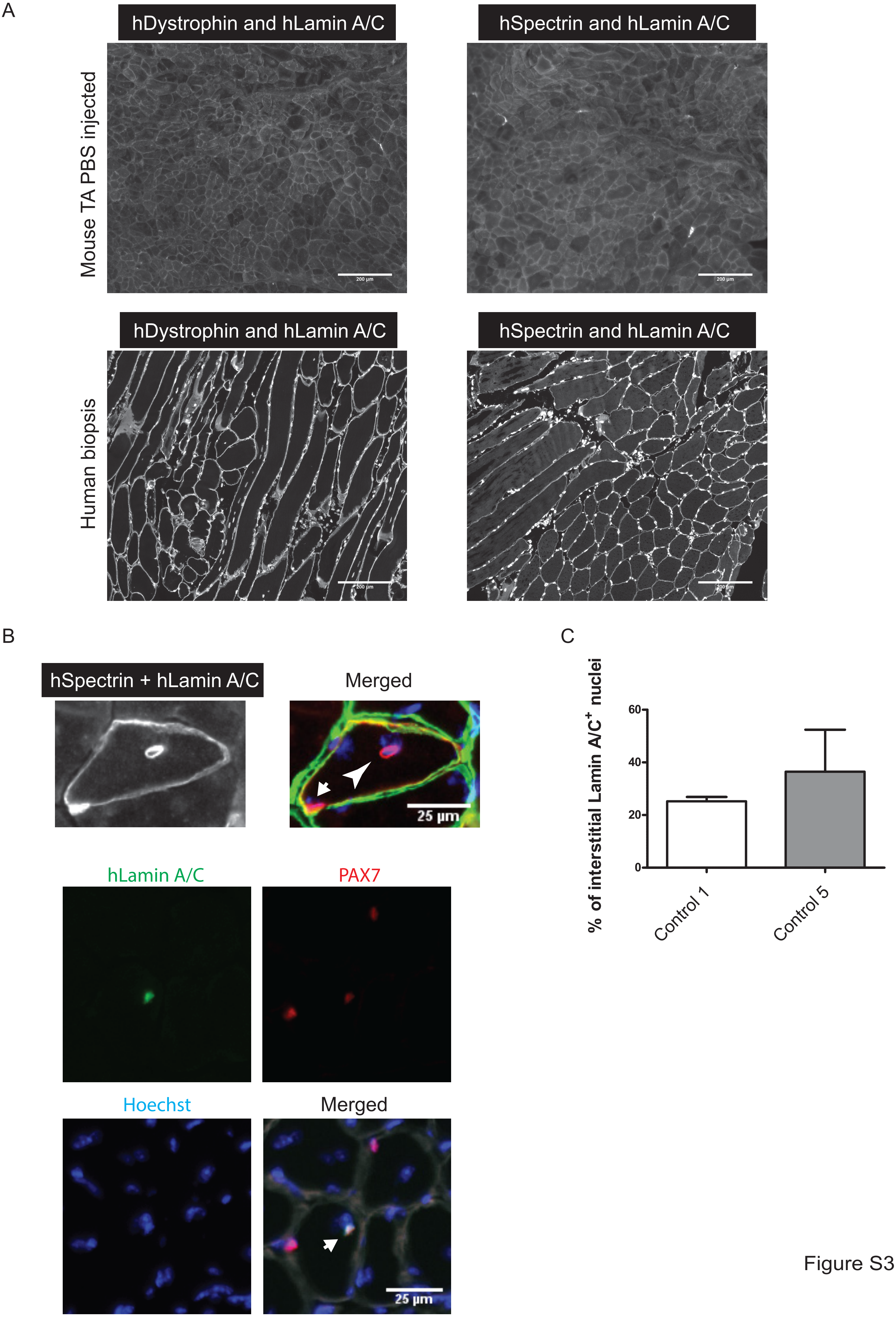
A
hDystrophin and hLamin A/C
hSpectrin and hLamin A/C
Mouse TA PBS injected
hDystrophin and hLamin A/C
hSpectrin and hLamin A/C
Human biopsis
B
hSpectrin + hLamin A/C
Merged
25 µm
hLamin A/C
PAX7
Hoechst
Merged
25 µm
C
% of interstitial Lamin A/C+ nuclei
60
40
20
0
Control 1
Control 5

Figure S3

SUPPLEMENTARY FIGURE LEGENDS

Figure S1 (related to Figure 1): Cell morphologies during differentiation of iPSCs into the myogenic lineage and after purification of myogenic progenitors. (A) Healthy-control iPSCs 3, 4, 5 and 6 were differentiated using a 35-day protocol consisting of GSK3β inhibition, FGF2 treatment, and a minimal medium (see Figure 7). Light microscope (4x magnification) images were taken before and during differentiation at the days indicated. Representative images are shown. (B) One day after FACS purification (described in Figure 7), light microscope images were taken from myogenic progenitors generated from healthy controls 4, 5 and 6 at a magnification of 10x and 20x. Representative images are shown.

Figure S2 (related to Figures 2 and 4): Differentiation, maturation of purified myogenic progenitors, and KEGG pathway analysis. (A) Myosin heavy chain (MHC) staining on 4 days differentiated myogenic progenitors from healthy controls 3- 8 and Pompe 3 – 7. For images of healthy controls 1 and 2, and Pompe 1 and 2, see van der Wal et al., 2017. Nuclei were stained with Hoechst. Images are representative for each differentiation. Fusion index during expansion was quantified and data are mean ± SD of 3 fields per point. (B) Staining of matured fibers from myogenic progenitors of healthy control 2. After 6-8 days of differentiation, cells were stained with Fast MHC, MHC, Titin and α-Actinin antibodies. Nuclei were stained with Hoechst and are shown in blue. (C) KEGG pathway analysis using DAVID of mapped genes comparing myogenic progenitors in proliferation phase (MPCs, this study) versus activated muscle stem cells (Charville et al., 2015). The 10 most significant pathways are shown.

Figure S3 (related to Figure 6): Positive and negative controls for analysis of *in vivo* engraftment and for cell contribution to muscle regeneration *in vivo*. (A) The upper panels show sections from the *tibialis anterior* from immunodeficient mice 4 weeks after injection with PBS only. The lower panel shows sections from a human biopsy of the quadriceps femoris. Sections were analyzed by immunohistochemistry using human specific Lamin A/C, Spectrin and Dystrophin antibodies (white). (B) Upper panel represent two different locations of Lamin A/C^+ nuclei within the same $Spectrin^+$ fiber. The human nuclei on a satellite cell position are indicated with an arrow and the myonuclei with an arrowhead. Lower panel shows a $PAX7^+$ (red), Lamin A/C^+ (green) nucleus in a $Laminin^+$ (grey) muscle fiber. (C) Percentage of Lamin A/C^+ nuclei present at the muscle interstitium per section of each biological replicate. Sections that showed engraftment were used for quantification. Data are mean ± SD (n= 2 TAs transplanted per line used. Each replicate was transplanted in different mice). All sections were counterstained with Hoechst (blue).

SUPPLEMENTARY TABLES

Table S1. Comparison of transgene-free skeletal-muscle differentiation protocols using GSK3β inhibition

	**Purification protocol**	**Fold expansion**	**Cryopreser vation**	**Duration**	**Fusion index**	**Engraftment**
**(Borchin et al., 2013)**	FACS	N.R.	N.R.	35 days	N.R.	N.R.
**(Xu et al., 2013)**	No purification, differentiation analysed in original plate	N.R.	N.R.	36 days	N.R.	Yes, unpurified culture
**(Shelton et al., 2014)**	No purification, differentiation analysed in original plate	N.R.	N.R.	50 days	N.R.	N.R.
**(Chal et al., 2015)**	No purification, differentiation analysed in original plate	N.R.	N.R.	50 days	N.R.	N.R.
**(Shelton et al., 2016)**	No purification, differentiation analysed in original plate	3x	N.R.	50 days	N.R.	N.R.

**(Choi et al., 2016)**	FACS	10^5x	Yes	30 days	10-15%	Yes, unpurified culture
**(Caron et al., 2016)**	Pre-plating	1250x	N.R.	26 days	N.R.	N.R.
**(Chal et al., 2016)**	Pre-plating	N.R.	Yes	35 days	N.R.	N.R.
**(Swartz et al., 2016)**	No purification, differentiation analysed in original plate	N.R.	Yes	36 days	N.R.	N.R.
**(Kim et al., 2017)**	No purification, differentiation analysed in original plate	N.R.	N.R.	50 days	N.R.	Yes, unpurified culture
**(van der Wal et al., 2017)**	FACS	5 x 10^7x	Yes	35 days	60-80%	N.R.
**This study**	FACS	5 x 10^{11}x	Yes	35 days	20-97%	Yes, purified culture

N.R: Not Reported

Table S2. Optimization of CHIR99021 concentration

Control 1

**CHIR99021**	**Days**	**Confluency**	**PAX7$^+$ cells**
3 µM	4	65%	15-10%
3 µM	5	50%	7-10%
3 µM	8	40%	1-2%
3 µM	10	40%	0%
4 µM	4	85%	30-35%
4 µM	5	100%	35-40%
4 µM	8	75%	30-35%
4 µM	10	50%	5-10%
5 µM	4	60%	15-20%
5 µM	5	70%	20-25%
5 µM	8	0%	0%
5 µM	10	0%	0%

Control 2

**CHIR99021**	**Days**	**Confluency**	**PAX7$^+$ cells**
3 µM	4	95%	1-2%
3 µM	5	100%	2-3%
3 µM	8	90%	3-4%
3 µM	10	95%	3-4%
4 µM	4	95%	10-15%
4 µM	5	95%	9-12%
4 µM	8	95%	10-15%
4 µM	10	85%	1-2%
5 µM	4	95%	3-5%
5 µM	5	95%	15-20%
5 µM	8	50%	7-10%
5 µM	10	30%	0%

Table S3. RNA sequencing datasets used in this study

Data source	Accession	Abbreviation	Reference
ENA	ERR975347	Activated Muscle Stem Cell 2	(Charville et al., 2015)
ENA	ERR975349	Activated Muscle Stem Cell 1 P38 treated 2	(Charville et al., 2015)
ENA	ERR975346	Activated Muscle Stem Cell 1	(Charville et al., 2015)
ENA	ERR975348	Activated Muscle Stem Cell 1 P38 treated 1	(Charville et al., 2015)
NCBI	GEO: *GSM3024344*	MPCs control 1A	This study
NCBI	GEO: *GSM3024345*	MPCs control 1B	This study
NCBI	GEO: *GSM3024346*	MPCs control 2A	This study
NCBI	GEO: *GSM3024347*	MPCs control 2B	This study
ENA	ERR975345	Quiescent Muscle Stem Cell 2	(Charville et al., 2015)
ENA	ERR975344	Quiescent Muscle Stem Cell 1	(Charville et al., 2015)
NCBI	GEO: *GSM3024348*	MPCs 4 days differentiated control 1A	This study
NCBI	GEO: *GSM3024349*	MPCs 4 days differentiated control 1B	This study
NCBI	GEO: *GSM3024350*	MPCs 4 days differentiated control 2A	This study
NCBI	GEO: *GSM3024351*	MPCs 4 days differentiated control 2B	This study
NCBI	GEO: *GSM2452280*	Neural stem cell 1	(McGrath et al., 2017)
NCBI	GEO: *GSM2452281*	Neural stem cell 2	(McGrath et al., 2017)
NCBI	GEO: *GSM2452282*	Neural stem cell 3	(McGrath et al., 2017)
ENCODE	ENCBS476ENC	Dermal Fibroblast 1	N/A
ENCODE	ENCBS459ENC	Mesenchymal stem cell 2	N/A
ENCODE	ENCSR828TEI	Primary Myotube 1	N/A
ENCODE	ENCBS018ENC	Chrondocyte 1	N/A
ENCODE	ENCLB014ZZZ	Cardiomyocyte	N/A
ENCODE	ENCBS460ENC	Mesenchymal stem cell 1	N/A
ENCODE	ENCSR000CUI	Myosatellite cell 2	N/A
ENCODE	ENCSR000AAG	Smooth muscle cell	N/A
NCBI	SRX689200	Primary hepatocytes 2	(Kambara et al., 2014)
ENCODE	ENCSR000CUI	Myosatellite cell 1	N/A
ENCODE	ENCBS019ENC	Chrondocyte 2	N/A
ENCODE	ENCBS475ENC	Dermal Fibroblast 2	N/A
ENCODE	ENCBS945YXY	Primary Kidney epithelial cell 2	N/A
NCBI	SRX673854	Primary hepatocytes 1	(Kambara et al., 2014)
ENCODE	ENCBS007YZP	Primary Kidney epithelial cell 1	N/A
ENCODE	ENCSR828TEI	Primary Myotube 2	N/A
ENCODE	ENCSR444WHQ	Primary Myoblast 2	N/A
ENCODE	ENCBS293AAA	Embryonic stem cell 1	N/A

ENCODE	ENCBS624XJG	Embryonic stem cell 2	N/A
ENCODE	ENCSR444WHQ	Primary Myoblast 1	N/A
ENCODE	ENCBS485ENC	Hematopoietic stem cell	N/A

Table S4. Antibodies and primers used in experiments

Name	Dilution or Sequence 5'-3'	Company	Assay
Mouse-anti-MF20	1:50	DSHB	IF
Rabbit-anti-Myogenin	1:100	Santa Cruz (sc-576)	IF
Mouse-anti-PAX7	1:100 or 1:20	DSHB	IF or IHC
Mouse-anti-α-Actinin	1:100	Sigma-Aldrich (A7811)	IF
Mouse-anti-Myosin (fast)	1:100	Sigma-Aldrich (M4276)	IF
Mouse-anti-Titin	1:50	DSHB	IF
Rabbit-anti-Laminin	1:100	Sigma-Aldrich (L9393)	IHC
Mouse-anti-hSpectrin	1:100	Leica (SPEC1-CE)	IHC
Mouse-anti-hDystrophin	1:100	Millipore (MABT827)	IHC
Mouse-anti-hLaminA/C	1:100	Vector Laboratories (VP-L550)	IHC
GAA Exon 1-2 fw	AAACTGAGGCACGGAGCG	IDTDNA	RT-qPCR
GAA Exon 1-2 rv	GAGTGCAGCGGTTGCCAA	IDTDNA	RT-qPCR
Set_1_fw	TTCCCAGGGCCGGTTAATGT	IDTDNA	PCR
Set_1_rv	GCTCTGGGCGGAGGAATATG	IDTDNA	PCR
Set_2_fw	CCTGAGTCCGGACCACTTTG	IDTDNA	PCR
Set_2_rv	CACCGGTTCAATTGCCGAC	IDTDNA	PCR
Set_3_fw	GTCTCTCACTCGGAAGGACAT	IDTDNA	PCR
Set_3_rv	TACCCCGAAGAGTGAGTTTGC	IDTDNA	PCR

SUPPLEMENTARY METHODS

GAA enzyme activity assay

Differentiated myogenic progenitors were harvested with ice-cold protein lysis buffer (50 mM Tris (pH 7.5)), 100 mM NaCl, 50 mM NaF, 1% Triton X-100 and one tablet Protease Inhibitor Cocktail cOmplete, with EDTA, (Roche, Penzberg, Germany) for 10 minutes on ice. GAA enzyme activity was measured as described previously (Kroos et al., 2007). Total protein concentrations were determined with the BCA protein assay kit (Pierce, Thermo Scientific, Waltham, MA).

qRT-PCR

qRT-PCR was measured with a CFX96 real-time system (Bio-Rad, Hercules, CA). cDNA was diluted 5x or 10x times and 4 µL was used in a qRT-PCR reaction consisting of a total volume of 15 µL with 7.5 µL iTaq Universersal SYBR Green Supermix (Bio-Rad, Hercules, CA), 10 pmol/µL forward and reverse primers (Table S4). Per plate, a standard curve was included with 5 dilutions.

Immunofluorescent analysis of *in vitro* differentiation

Myogenic progenitors were stained as described previously (van der Wal et al., 2017). Briefly, cells were permeabilized for 5 minutes with 0.1% Triton X-100 (AppliChem, Darmstadt, Germany) in PBS and blocked for 30 minutes at room temperature in blocking solution (PBS-T (0.1% Tween, Sigma-Aldrich, Irvine, UK) with 3% BSA (Sigma-Aldrich, Irvine, UK)). Primary antibodies (Table S4) were incubated for 1 hour at room temperature and diluted into 0.1% BSA in PBS-T, washed with PBS-T and incubated with secondary antibodies (1:500, Alexa-Fluor-488-α-mouse, A11001, Alexa-Fluor-594-α-rabbit, A10474, Alexa-Fluor-488-α-rabbit,

A11008, Invitrogen, Carlsbad, CA; or horse anti-mouse biotin, BA-2000, Vector Laboratories, Burlingame, CA). When a secondary biotinylated antibody was used, cells were washed three times for 5 minutes with PBS-T and incubated with Streptavidine 594 (1:500, S-32356, Invitrogen, Carlsbad, CA,). The cells were subsequently washed two times for 5 minutes with PBS and incubated for 15 minutes with Hoechst (1:15000, Thermo Scientific, Waltham, MA) before imaging.

Generation of induced pluripotent stem cells
Control iPSC lines were previously reprogrammed, characterized and cultured as described in van der Wal et al. (van der Wal et al., 2017). Healthy control 3 and healthy control 4 iPSCs were a gift from Dr. Mehrnaz and Prof. Joost Gribnau . Healthy control 2 (previously characterized in (Dambrot et al., 2013)), healthy control 5 (LUMC0004iCTRL10), and healthy control 8 (LUMC0030iCTRL12) iPSCs were gifts from Dr. Christian Freund and Prof. Christine Mummery. Using the MycoAlert™ Mycoplasma Detection Kit (Lonza, Walkersville, MD), the iPSC lines were regularly tested for contamination with mycoplasma. All results in this study were obtained with cultures that had tested negative. The identities of cell lines used in this study were confirmed by DNA sequencing.

Generation and expansion of myogenic progenitors from iPSCs
iPSC cultures in 100 mm dishes were used to initiate myogenic differentiation as described previously (van der Wal et al., 2017). Briefly, after 5 days of iPSC expansion, differentiation into myogenic progenitors was started with myogenic differentiation medium (DMEM/F12, 1% Insulin-Transferrin-Selenium-Ethanolamine (ITS-X), 1% penicillin/streptomycin/L-glutamine (P/S/G), all Gibco, Waltham, MA) supplemented with 3.5 µM CHIR99021 (Axon Medchem, Groningen, the Netherlands) for 5 days; and changed to myogenic differentiation medium supplemented with 20 ng/ml FGF2 (Prepotech, Rocky Hill, NJ) for 14 days. For the last 16 days, cells were cultured in myogenic differentiation medium only. Myogenic progenitors were purified using FACS with anti-C-MET-APC (1:50, R&D systems, Minneapolis MN), and anti-HNK-1-FITC (1:100, Aviv Systems Biology, San Diego, CA) antibodies; and Hoechst (33258, Life Technologies, Carlsbad, CA) was added to stain live cells. The c-MET^{+}/Hoechst^{+}/Hnk-1^{-} fraction was sorted in myogenic progenitor proliferation (MMP) medium (DMEM high-glucose supplemented with 10% fetal bovine serum, 1% penicillin/streptomycin/L-glutamine and 100 ng/ml FGF2) supplemented with 1x Revitacell supplement (Gibco, Waltham, MA) on ECM (Sigma-Aldrich, E6909)-coated dishes as described (van der Wal et al., 2017).

SUPPLEMENTARY REFERENCES

Borchin, B., Chen, J., and Barberi, T. (2013). Derivation and FACS-mediated purification of PAX3+/PAX7+ skeletal muscle precursors from human pluripotent stem cells. Stem Cell Reports *1*, 620-631.

Caron, L., Kher, D., Lee, K.L., McKernan, R., Dumevska, B., Hidalgo, A., Li, J., Yang, H., Main, H., Ferri, G., *et al.* (2016). A Human Pluripotent Stem Cell Model of Facioscapulohumeral Muscular Dystrophy-Affected Skeletal Muscles. Stem Cells Transl Med *5*, 1145-1161.

Chal, J., Al Tanoury, Z., Hestin, M., Gobert, B., Aivio, S., Hick, A., Cherrier, T., Nesmith, A.P., Parker, K.K., and Pourquie, O. (2016). Generation of human muscle fibers and satellite-like cells from human pluripotent stem cells in vitro. Nat Protoc *11*, 1833-1850.

Chal, J., Oginuma, M., Al Tanoury, Z., Gobert, B., Sumara, O., Hick, A., Bousson, F., Zidouni, Y., Mursch, C., Moncuquet, P., *et al.* (2015). Differentiation of pluripotent stem cells to muscle fiber to model Duchenne muscular dystrophy. Nat Biotechnol *33*, 962-969.

Charville, G.W., Cheung, T.H., Yoo, B., Santos, P.J., Lee, G.K., Shrager, J.B., and Rando, T.A. (2015). Ex Vivo Expansion and In Vivo Self-Renewal of Human Muscle Stem Cells. Stem Cell Reports *5*, 621-632.

Choi, I.Y., Lim, H., Estrellas, K., Mula, J., Cohen, T.V., Zhang, Y., Donnelly, C.J., Richard, J.P., Kim, Y.J., Kim, H., *et al.* (2016). Concordant but Varied Phenotypes among Duchenne Muscular Dystrophy Patient-Specific Myoblasts Derived using a Human iPSC-Based Model. Cell Rep *15*, 2301-2312.

Dambrot, C., van de Pas, S., van Zijl, L., Brandl, B., Wang, J.W., Schalij, M.J., Hoeben, R.C., Atsma, D.E., Mikkers, H.M., Mummery, C.L., *et al.* (2013). Polycistronic lentivirus induced pluripotent stem cells from skin biopsies after long term storage, blood outgrowth endothelial cells and cells from milk teeth. Differentiation *85*, 101-109.

Kambara, H., Niazi, F., Kostadinova, L., Moonka, D.K., Siegel, C.T., Post, A.B., Carnero, E., Barriocanal, M., Fortes, P., Anthony, D.D., *et al.* (2014). Negative regulation of the interferon response by an interferon-induced long non-coding RNA. Nucleic Acids Res *42*, 10668-10680.

Kim, J., Magli, A., Chan, S.S.K., Oliveira, V.K.P., Wu, J., Darabi, R., Kyba, M., and Perlingeiro, R.C.R. (2017). Expansion and Purification Are Critical for the Therapeutic Application of Pluripotent Stem Cell-Derived Myogenic Progenitors. Stem Cell Reports *9*, 12-22.

Kroos, M.A., Pomponio, R.J., Hagemans, M.L., Keulemans, J.L., Phipps, M., DeRiso, M., Palmer, R.E., Ausems, M.G., Van der Beek, N.A., Van Diggelen, O.P., *et al.* (2007). Broad spectrum of Pompe disease in patients with the same c.-32-13T->G haplotype. Neurology *68*, 110-115.

McGrath, E.L., Rossi, S.L., Gao, J., Widen, S.G., Grant, A.C., Dunn, T.J., Azar, S.R., Roundy, C.M., Xiong, Y., Prusak, D.J., *et al.* (2017). Differential Responses of Human Fetal Brain Neural Stem Cells to Zika Virus Infection. Stem Cell Reports *8*, 715-727.

Shelton, M., Kocharyan, A., Liu, J., Skerjanc, I.S., and Stanford, W.L. (2016). Robust generation and expansion of skeletal muscle progenitors and myocytes from human pluripotent stem cells. Methods *101*, 73-84.

Shelton, M., Metz, J., Liu, J., Carpenedo, R.L., Demers, S.P., Stanford, W.L., and Skerjanc, I.S. (2014). Derivation and expansion of PAX7-positive muscle progenitors from human and mouse embryonic stem cells. Stem Cell Reports *3*, 516-529.

Swartz, E.W., Baek, J., Pribadi, M., Wojta, K.J., Almeida, S., Karydas, A., Gao, F.B., Miller, B.L., and Coppola, G. (2016). A Novel Protocol for Directed Differentiation of C9orf72-Associated Human Induced Pluripotent Stem Cells Into Contractile Skeletal Myotubes. Stem Cells Transl Med *5*, 1461-1472.

van der Wal, E., Bergsma, A.J., van Gestel, T.J.M., In 't Groen, S.L.M., Zaehres, H., Arauzo-Bravo, M.J., Scholer, H.R., van der Ploeg, A.T., and Pijnappel, W. (2017). GAA Deficiency in Pompe Disease Is Alleviated by Exon Inclusion in iPSC-Derived Skeletal Muscle Cells. Mol Ther Nucleic Acids *7*, 101-115.

Xu, C., Tabebordbar, M., Iovino, S., Ciarlo, C., Liu, J., Castiglioni, A., Price, E., Liu, M., Barton, E.R., Kahn, C.R., *et al.* (2013). A zebrafish embryo culture system defines factors that promote vertebrate myogenesis across species. Cell *155*, 909-921.
